# Transposed Letter Priming Effects and Allographic Variation in Arabic: Insights From Lexical Decision and the Same–Different Task

**DOI:** 10.1037/xhp0000621

**Published:** 2019-06

**Authors:** Sami Boudelaa, Dennis Norris, Abdesattar Mahfoudhi, Sachiko Kinoshita

**Affiliations:** 1 Department of Linguistics, United Arab Emirates University, and Department of Psychology, University of Cambridge; 2 MRC Cognition and Brain Sciences Unit, Cambridge, United Kingdom; 3 Department of English Literature, Australian College of Kuwait, and Centre for Child Evaluation and Teaching, Kuwait; 4 Department of Cognitive Science, Macquarie University

**Keywords:** TL priming, Allography, same-different matching task

## Abstract

Reading is resilient to distortion of letter order within a word. This is evidenced in the “transposed-letter (TL) priming effect,” the finding that a prime generated by transposing adjacent letters in a word (e.g., jugde) facilitates recognition of the base word (e.g., JUDGE), more than a “substituted-letter” control prime in which the transposed letters are replaced by unrelated letters (e.g., junpe -JUDGE). The TL priming effect is well documented for European languages that are written using the Roman alphabet. Unlike these languages, Arabic has a unique position-dependent allography whereby some letters change shape according to their position within a word. We investigate the TL priming effect using a lexical decision (Experiment 1) and a same–different match task with Arabic words (Experiment 2) and nonwords (Experiment 3). No TL priming effects were found in Experiment 1, suggesting that the lexical-decision task engages lexical access processes that are sensitive to the Semitic nonlinear morphological structure. Experiments 2 and 3 revealed a robust TL priming effect overall. Nonallographic TL primes produced significantly larger facilitation than allographic TL primes, indicating that Arabic readers use allographic variation to resolve the uncertainty in letter order during the early stages of orthographic processing. The implication of these results for current letter position coding models is discussed.

## Public Significance Statement

Transposed-letter (TL) priming effects are popularly interpreted as reflecting noisy perception of letter order. The Arabic writing system has a unique allographic feature whereby a letter’s shape and letter spacing depend on its position within a word. We show that TL priming effects for Arabic words and nonwords are modulated by allography in the same–different task. It is important to consider which unique property of the language/writing system produces the cross-language variation, and how it does so.

Much of the recent cognitive and neurocognitive research on reading has come to focus on the front end of the mechanisms underpinning visual word recognition, namely the recognition of letters comprised by a word (e.g., Bick, Goelman, & Frost, 2011; Dehaene, Cohen, Sigman, & Vinckier, 2005; Gómez, Ratcliff, & Perea, 2008; Grainger, Rey, & Dufau, 2008; Norris & Kinoshita, 2012a). Three issues are at the center of this research. The first relates to the existence of abstract letter representations and how these serve as targets for the visual input perceived during reading. The second concerns the way in which information about letter order is coded, and the final issue relates to whether different languages bring different constraints to bear on the question of letter abstractness and letter-order coding.

Words can be written in letters of different sizes, different fonts, and different cases. Does this low-level perceptual variation play a role in reading or do the representations that drive lexical access abstract over these perceptual variations? From studies using the masked priming paradigm (Forster & Davis, 1984) there is now a wealth of evidence that reading is mediated by abstract letter representations. In this paradigm, a prime is presented briefly (typically 30–60 ms) following a forward mask consisting of uninformative hash signs and backward masked by the target to which an overt response is required. Responses to the target are facilitated when the prime is related to the target. Using masked priming with a lexical-decision task, Bowers, Vigliocco, and Haan (1998) showed that lowercase primes that were visually dissimilar to the targets presented in uppercase (e.g., *edge-EDGE*) yielded comparable facilitation effects to those produced by lowercase primes that were visually similar to their targets (e.g., *kiss-KISS*). This result has been taken as evidence that the letter representations supporting visual word recognition are abstract. Kinoshita and Kaplan (2008), and Kinoshita and Norris (2009) extended this finding to single letters and nonwords showing that the involvement of abstract letter representations is not specific to reading words. They used the same–different matching task in which a reference stimulus is presented in advance of the prime-target sequence and the subject’s task is to decide whether the target is the same as or different from, the reference. The size of priming did not differ between visually similar prime-target pairs (e.g., *c/C, x/X*) and visually dissimilar pairs (e.g., *b/B, e/E*).^1^

The second issue concerns how letter order is coded. Recent research has amassed considerable empirical evidence showing that readers can cope with violations in the canonical order of letters in a word. This claim is supported by the phenomenon called the transposed-letter (TL) priming effect. This refers to the finding that a nonword prime constructed by transposing two adjacent word-internal letters (e.g., *jugde*) facilitates the recognition of the base word (*JUDGE*) more than a substituted-letter (SL) control prime in which the transposed letters are replaced by unrelated letters (e.g., junpe; e.g., Perea & Lupker, 2003). As with the data showing evidence for abstract letter identities, TL priming effects are also found with nonword targets in the same–different match task (Kinoshita & Norris, 2009). According to the noisy position view (Gómez et al., 2008; Norris & Kinoshita, 2012a), TL priming effects reflect the uncertainty in letter order arising from noise in the early stages of visual perception. Specifically, within the brief time that the prime is presented, the spatial order of neighboring letters in a letter string is ambiguous (i.e., it is uncertain whether *g* in *jugde* is to the left or right of *d*). To the extent that there is some possibility that ‘d’ precedes ‘g,’ the TL prime *jugde* will match *JUDGE* to a greater degree than the control prime *junpe*.

The evidence from the research summarized above strongly suggests that the visual word recognition system operates with abstract letter representations and that it shows a degree of tolerance in the processing of letter order. Most of this evidence has come from European languages that are written using the Roman alphabet. This brings us to the third issue driving research into visual word recognition, which relates to whether the structural characteristics of different languages can influence theorizing about the nature of the representation of letters subserving the reading process. Previous cross-linguistic research strongly suggests that typologically different languages not only organize their lexical spaces differently, but they also seem to weight different domains of linguistic knowledge differently (e.g., Frost, 2012 for Hebrew; Lee & Taft, 2011 for Korean). More specifically, it has been claimed that reading in languages like Hebrew and Arabic is subject to an extreme letter-coding scheme such that transposing the letters of a prime item does not generate the strong facilitation of target recognition typical in Indo-European languages. This claim is mainly based on the consistent absence of Transposed Letter (TL) priming in lexical decision with Hebrew (e.g., Velan, Deutsch, & Frost, 2013; Velan & Frost, 2009, 2011), and Arabic (e.g., Perea, Abu Mallouh, & Carreiras, 2010), which contrasts sharply with the facilitatory effects found in languages written in the Roman alphabet. What is the origin of this cross-linguistic difference?

As we will see in more detail below, written Arabic has two unique features that distinguish it from European languages written in the Roman alphabet: One is its Semitic morphological structure, the second is its extensive position-dependent allography. There are reasons to suspect that these two features may operate at different levels or stages of visual word recognition, and for this reason we will use two tasks that are differentially sensitive to stages of orthographic and lexical processing. Specifically, in the first experiment, we use the lexical-decision task to engage lexical processing and to confirm the absence of the TL priming effects in this task, as has been reported consistently with Semitic languages. This experiment also serves to validate our stimuli. We then use these stimuli in the same–different match task, in which the orthographic priming effects have been shown to operate at the level of prelexical orthographic representations consisting of abstract letter identities (Kinoshita & Norris, 2009; Kinoshita, Gayed, & Norris, 2018), and to be insensitive to morphological structure in European languages written in the Roman alphabet (Duñabeitia, Kinoshita, Carreiras, & Norris, 2011). Specifically, we focus on the orthographic system of Modern Standard Arabic (MSA), with its unique characteristics, to investigate whether the TL priming effect, which has been used as the main marker of letter order coding flexibility, is modulated by allographic variation. In what follows, we briefly present the key characteristics of the MSA orthographic system, then we summarize previous experimental research that addressed orthographic processing in Arabic.

## The MSA Orthographic System

The MSA orthographic system (see Daniels, 2013) operates with 28 letters that are written cursively from right to left. From the visual point of view, these letters fall into nine groups as shown in Table 1.[Table-anchor tbl1]

Groups 1 to 8 consist of letters with exactly the same base shape save for the presence of dots. Thus in Group 1, for instance, the three letters share the same base shape, but differ with respect to the number and position of dots, with in particular, the letter ب /b/ exhibiting a single dot underneath it, while the ت /t/, and ث /θ/, respectively, feature two and three dots above them. The exception to this pattern is the 10 letters of Group 9 which have unique shapes that they do not share with each other or with any other letter.

A number of orthographic characteristics make MSA an ideal writing system to investigate the issues of abstract letter representations and letter order coding. One relates to the allographic nature of the script whereby each letter can take as many as four different shapes depending on its position within the word: an initial, a medial, a final, and an isolated form as illustrated in Table 2.[Table-anchor tbl2]

Two variables interact to determine which allograph is used for a given letter. These are the position of the letter in the word, initial, medial, or final, and the presence or absence of a ligature with the preceding letter. Twenty-two of the 28 letters are fully ligating letters (i.e., they connect to the preceding and the following letter) and have four allographs, whereas the remaining six are partially ligating letters (i.e., they ligate only to the preceding letter) and have only two allographs each.

One consequence of the interaction of these two variables is that besides triggering changes in the form of a letter (e.g., لعب – لبع*^2^ ‘*play*’), allography manifests itself in two other ways. First, it can appear as a change in ligation pattern as in أفشلنا - أشفلنا* ‘*fail*,’ where the underlined letter changes from a letter ـشـ that ligates to the left and right to a شـ ligating only to the left. Second, allography can show up as a redistribution of blank-spaces within the word. For instance, the blank space (indicated by the underscore here for ease of illustration) comes after the second letter in مو_لد *birth*, but after the third letter in the transposed nonword ملو_د. Importantly, although change in ligation patterns can occur without redistribution of blank spaces within the word (e.g., ألهم - أهلم* ʔ*lhm*-ʔ*hlm* ‘*inspiring*’), the reverse is not true in the sense that the redistribution of blank-spaces is always accompanied by a change in ligation patterns (e.g., أذ_هب - أهذ_ب ʔ*ðhb-ʔhðb* ‘*going-civilize*’) a change in letter form (e.g., أعلم - ألعم ʔ*ʕlm- ʔlʕm* ‘*inform-uncle*’) or both (e.g., أعد_م – أعمد ʔ*ʕdm-ʔʕmd* ‘*execute-intend*’). Furthermore, a change in letter form does not necessarily trigger a blank-space redistribution and vice versa. In the transposed letter item يتطعلا_ن* the shape of the letters has changed compared with the original form يتطلعا_ن ‘*look forward to*,’ but the blank space remains in the same position, after the sixth letter, in both items. For the present purposes, we have opted to treat the three manifestations of allography as equivalent. We do this for two reasons, first, because a change in the form of the letter, a change in its ligation pattern, or the redistribution of blank spaces within the word may provide equally important cues about the identity of the letters within the orthographic string, and about its length, and second, because we are interested in the change in the overall shape of the word, which is equally apparent when the shape of the letter or its ligation pattern changes, or indeed when the blank spaces within the word are redistributed. In addition to this, the constraints we had to observe when building the experimental materials made it impossible to covary for instance letter-form change and ligation change without creating an existing root, which we strove to avoid. We will nevertheless consider the potential contribution of the different forms of allography in our post hoc statistical analyses.

In sum, the critical feature of MSA is that allography, in all its manifestations, is highly informative with regards to letter position in two important ways. First, when a reader sees the allograph ـعـ, for instance, they know that it is neither at the beginning nor end of the word, whereas seeing the allograph ع is an unequivocal cue that it is at the end of the word. Second, the different allographs of a letter can affect the location of blank spaces within the orthographic sequence especially when the letter is nonligating. Note for instance that the blank space, indicated by an underscore, after the third letter, in the base word تجر_بة *tjr_bt* ‘*experience*’ appears after the second letter in the transposed nonword version تر_جبة *tr_jbt*. The sort of information conveyed by the various forms of the letters in MSA and the accompanying redistribution of the location of blank spaces in the orthographic sequence are qualitatively very different from the allography in the Roman script (uppercase and lowercase letters) which neither depends on letter position nor alters the location of blank spaces within a word.^3^ Against this background, the question we address here is whether readers of MSA can take advantage of the order constraints provided by the position-specific allographs, or instead rely entirely on a more abstract level of letter representation that discards the allographic detail.

Another equally important feature of MSA orthography is that it is a predominantly consonantal system all of whose letters represent consonant phonemes except for three, known as *matres lectionis*, which serve to indicate the three long vowels of the language /aa, uu, ii/. The remaining three short vowels /a, u, i/ have no corresponding letters; instead they are written as diacritical marks above or below the letter. This characteristic of the script is intimately linked to the Semitic morphological system which relies on a clear distinction between two morphemes: the root, typically made up of thee consonants, and the word pattern (WP) essentially made up of vowels and a subset of consonants. The root provides constraining semantic information and plays an earlier and more durable role in the lexical access process than the word pattern which is interleaved with the root and conveys morpho-syntactic and phonological information, affecting the processing dynamics only during a limited time window (Boudelaa & Marslen-Wilson, 2005). Given the salience of the consonantal root as an access and representational unit in lexical processing in Arabic (Boudelaa & Marslen-Wilson, 2004, 2005, 2011, 2015), the question arises of how the transposition or substitution of root consonants modulates TL-priming effects.

## Previous Research on Orthographic Processing of Arabic

Perea et al. (2010) and Carreiras, Perea, Gil-López, Abu Mallouh, & Salillas (2013) have spearheaded research into letter order coding and abstract letter identities in Arabic. Using both lexical (lexical decision) and nonlexical (same–different matching) tasks, these authors have collectively made two important claims. One is that the Semitic morphological structure, comprising the tri-consonantal root and a word pattern, plays a key role in the early orthographic processing in Arabic. Velan and Frost (2009, 2011) have previously shown using a lexical task (lexical decision) that in Hebrew, another Semitic language, TL priming effects are absent when the transposed letters are root consonants, and argued that this reflects a Semitic orthographic structure that is fundamentally different from European languages. Perea et al. (2010) replicated this finding with Arabic and extended it to the same–different task (Perea, Abu Mallouh, Garcı A-Orza, & Carreiras, 2011, Experiment 1). The authors took the latter as evidence that the Semitic morphological structure modulates prelexical orthographic processing, arguing that the masked priming same–different task is sensitive to morphology. However, Kinoshita, Norris, and Siegelman (2012) questioned this claim, noting that Perea et al. examined TL effects without taking into account allographic variations, using primes that appear with different allographs when their root letters are transposed (e.g., بعيد - عبيد *bʕiid-ʕbiid* ‘*far-slaves*’; the differing allographs are underlined in the MSA script). In contrast to Perea et al.’s (2011) finding for Arabic, Kinoshita et al. (2012), using Velan and Frost’s (2011) stimuli in Hebrew, which has much more limited position-dependent allography, found robust TL priming effects that were not modulated by morphology in the same–different task. Thus, it remains to be seen whether the finding reported by Perea et al. (2011) for Arabic is replicable when allographic variation is controlled.

The second main claim made by these authors is that allographic variation plays little or no role in visual word recognition in Arabic. This claim is based on studies (Carreiras, Perea, & Abu Mallouh, 2012, 2013) that used single letters in the same–different match task to compare identity priming effects with nonallographic, that is with no change in letter shape (e.g., د, د /d/), and allographic (e.g., ع, ـعـ /ʕ/) variants of the same letter. The authors reported priming effects of the same magnitude for allographic and nonallographic letter pairs, much like the finding regarding visually similar and dissimilar uppercase-lowercase letter pairs in the Roman alphabet (Kinoshita & Kaplan, 2008; Norris & Kinoshita, 2008), and concluded that “priming of abstract letter representations is a universal phenomenon” (Carreiras et al., 2012, p. 685). A limitation of this claim, however, is that allographic variation in Arabic is informative in the context of the word (a sequence of letters): the allograph ـــعـ tells the reader that there is a letter preceding it and a letter following it, and that both are ligating letters. Presented as a single letter, this allograph says nothing about position within a word, and so it is not too surprising that the Arabic allographic variation functions just like the uppercase and lowercase variations in the Roman alphabet.

The recent study by Perea, Abu Mallouh, and Carreiras (2013) aimed to assess the role of visual similarity using a masked priming lexical-decision task with 3rd and 6th graders as well as adult readers. The authors substituted either a letter in a prime having the same visual form (i.e., the same allograph in terms of the ligation pattern between a word’s letters) as the target word (e.g., كتزب - كتاب “ktzb”–“ktaab”), or a letter having a different visual form from the target as a result of ligation (كتخب - كتاب *ktaab-ktxb*). The size of the priming did not differ between these two types of primes in either developing readers or adult readers. Given the parallel with the visual similarity manipulation carried out with the lowercase prime-uppercase target pairs in the Roman alphabet (e.g., *edge-EDGE* vs. *kiss-KISS*, Bowers et al., 1998), Perea et al. concluded that allographic variation plays little role in Arabic. Note however that the visual similarity manipulation concerned a nonroot letter, and that the prime and target always shared the three letters of the root {ktb}. From the point of view that lexical processing in Arabic is tuned to picking up root consonants (e.g., Boudelaa, 2014; Boudelaa & Marslen-Wilson, 2005, 2015), there is little reason to expect the two conditions to behave differently. That is, the message from this experiment may not be that visual form has no role to play, but that root priming in Arabic is robust and can be observed in both adult and developing readers.

The conclusion drawn by these authors that allography does not modulate visual word recognition processes in Arabic seems inconsistent with recent data concerning readers of MSA who have Letter Position Dyslexia (Friedmann & Haddad-Hanna, 2012). These dyslexic readers have a selective deficit in letter position encoding manifested in a disproportionate number of reading errors involving letter migrations within words. The point of interest is that their letter-migration errors are modulated by position-dependent allography. They made 85% letter migration errors with words involving no allographic variation (i.e., words comprising the same letter allograph in the base word and TL word, e.g., تمهل - تهمل *tmhl-thml* ‘*give notice-neglect*’); in contrast, words that would involve allographic variation when their letters are transposed (e.g., جاهز - جهاز *jaahz-jhaaz* ‘*ready-device*’) hardly elicited any errors. This suggests that allographic information is indeed encoded and is used to resolve uncertainty in letter position. What is unclear, however, is whether the use of letter-form information is characteristic of an immature reading system specific to individuals with dyslexia and does not apply to normal skilled readers, as suggested by Carreiras et al. (2012, p. 690).

The absence of allographic effects in Arabic as claimed by Perea et al. (2013) and Carreiras et al. (2012) is equally at variance with data from Uyghur, an agglutinative non-Semitic language from the Turkic family that uses an Arabic-based script in which both consonants and vowels are fully specified (Yakup, Abliz, Sereno, & Perea, 2014, 2015). Using a Rapid Serial Visual Presentation (RSVP) with sentences containing jumbled words, in which the internal letters were rearranged in a way that either did or did not lead to allographic changes, Yakup et al. (2014) showed that response accuracy was significantly lower for target words in jumbled sentences involving a letter shape change, thus suggesting that position-dependent allography affects letter position coding in Uyghur. Similar results were found by the same authors using a masked priming lexical decision in which it was found that transposed and substituted letter primes that did not incur any allographic changes showed significantly more facilitation than those that did (Yakup et al., 2015).

In summary, then, the Arabic dyslexic data and the Uyghur data for which strong effects of allography have been reported are at variance with the masked priming data reported by Perea et al. (2011, 2013) and Carreiras et al. (2012), which did not find any modulatory effects of priming by allography. Given the methodological shortcomings of the Arabic masked priming studies described above, however, the role of position-dependent allography in visual word recognition in intact readers of Arabic remains to be determined.

In the present study, we addressed this issue by evaluating the extent to which allographic variation can modulate TL priming effects in Arabic. To ascertain the locus of these effects, we evaluated the effects allography has on TL priming using the masked priming lexical-decision task (Experiment 1), the same–different matching with existing Arabic words (Experiment 2), and grapho-tactically legal nonwords (Experiment 3).

## Experiment 1: Masked Priming Lexical Decision

Lexical decision is a classic lexical task in which the participant is presented with letter strings that are either real words or word-like nonwords. In this task, the subject has to verify whether each item is a word or not. This task is standardly thought to engage lexical access processes and to be sensitive to the way lexical space is organized in a given language. We elected to use this task in the first experiment because it naturally complements the same different matching task that we will use in Experiments 2 and 3. Furthermore, the lexical-decision task, but not the same–different task, requires lexical access. In the Bayesian Reader framework (Norris, 2006; Norris & Kinoshita, 2012a), in the lexical-decision task the participant has to match the input against the whole lexicon, whereas, in the same–different task, they have to match it against a single item: the referent (Norris & Kinoshita, 2008). This means that the characteristics of lexical space such as neighborhood density and morphological structure should modulate priming in lexical decision but not in the same–different match task. Accordingly, if the specific characteristics of the Arabic lexicon come into play in lexical decision, but not in the same–different match task, then we expect to see different TL priming effects in Experiment 1 compared with Experiments 2 and 3. A second reason for using lexical decision is that there has been no systematic study of orthographic priming effects in Arabic controlling for allographic variation with transposed letter primes in this task. Finally, lexical decision is a ubiquitously used task in the orthographic priming literature looking at transposed letter effects and, as such, it will allow us to maintain comparability with previous research.

In past research using lexical decision, Velan and Frost (2009, 2011) consistently found no TL priming effects for Hebrew native words with the typical Semitic morphological structure. Perea et al. (2010) extended these results to Arabic, using transposed-letter Arabic words as primes for existing word target (e.g., مقعد – معقد *mqʕd-mʕqd*, ‘*seat-complex*’) and found no TL priming effects; they thus concluded that “the order of the root letters is allowed only a minimum degree of perceptual noise to avoid activating the wrong root” (pp. 375, 378). The present experiment extended these studies in two respects. First, it used real word targets paired with transposed and substituted letter nonword primes comprising nonexisting roots. This allowed us to establish whether the absence of TL priming effects reported by Perea et al. (2010) was attributable to competition among existing roots, or genuinely reflected the processing dynamics engaged during lexical decision in this language. Second, and more importantly, we added the manipulation of allography such that when two consonants of the base root were transposed or substituted, this either caused a change in the shape of the manipulated letter and/or brought about a redistribution of blank space locations. This variable was not controlled in the Perea et al. (2010, 2011), and as a consequence it is possible that the absence of TL effects when the root letters were manipulated in Arabic could in part be due to this confounding factor.

### Method

#### Participants

Fifty-nine participants were randomly selected from the female campus of the United Arab Emirates University to take part in this experiment. Their age ranged between 20 and 24 years old, and they all were native speakers of dialectal Emirati Arabic, used Modern Standard Arabic on a daily basis, and had normal or corrected-to-normal vision.

The sample size was guided by, and is much greater than, previous studies of TL priming lexical decision experiments in Arabic (Perea et al., 2010: 26 participants in Experiment 1, 28 participants in Experiment 2). More generally, the sample size in each of the three experiments reported here provided an estimated power greater than 0.9 to detect the effects of interest if they existed.

#### Stimuli and design

We selected 60 words to be used as targets for the ‘word’ response condition. They ranged between 4 and 8 letters in length (mean 6.12 letters), with an average frequency of 15.36 per million (range: .01–234) in the Aralex database (Boudelaa & Marslen-Wilson, 2010). Each of the words was preceded by one of six types of prime (see Table 3). The first was an Identity prime (e.g., يسعدون - يسعدون *ysʕduun-ysʕduun* ‘*be happy-be happy*), and the sixth was the all-letter-different baseline nonword prime, used to establish that the task is sensitive to the masked priming manipulation (e.g., تتناثج * *ttnaaθj*). The second to fifth prime types involved the two critical manipulations, Type of Change (letter transposition vs. substitution) and Allography, crossed orthogonally. All primes were legal nonwords. In Condition 2, two letters of the TL−Allog primes were transposed without causing any allographic changes (e.g. يسعدون - يعسدون *yʕsduun-ysʕduun ‘*be happy*’). In contrast, in Condition 3, TL+Allog, two letters of the prime were transposed so as to undergo allographic changes (e.g., يسدعون - يسعدون *ysdʕuun-ysʕduun ‘*be happy*’). Note for example the change of the underlined letter ʕ in the Arabic-script example from the allograph ــعــ when flanked by two ligating letters in the base word to the allograph عــ when the preceding letter does not ligate in the prime. Note further that the blank spaces appear after letters 3 and 5 in the TL+Allog prime, but after letters 4 and 5 in the target. More specifically, the allographic changes in this condition consisted of (a) changes in the shape of the letter (e.g., ع to ـعـ or ل to ـلـ) 48.81% of the time, (b) changes in ligation of the letters (e.g., ر to ـر or خـ to ـخـ) 9.66% of the time, and (c) changes in ligation and blank space location (e.g., ينذ_ر_و_ن – يذ_نر_و_ن) 41.53% of the time. In Condition 4, SL−Allog, two letters of the target were substituted to create a prime without any allographic changes. In particular, one of the substituted letters was from the same Letter group as the original letters 56.78% of the cases (e.g., يشغدون* – يسعدون *yʃɣduun-ysʕduun ‘*be happy*’). In the remaining 43.22% of the items, the substitution letters were from different Letter groups that nonetheless preserved the ligation pattern and blank space location of the base word (e.g., ينكفقا_ن* – ينطلقا_ن *ynkfqaan- ynt^ʕ^*lqaan* ‘*start off*’). In Condition 5, labeled SL+Allog, two letters of the target were substituted so as to cause allographic changes in the prime (e.g., يسزرون* - يسعدون*yszruun-ysʕduun ‘*be happy*’). These changes pertained to letter shape in 1.69% of the items, to letter ligation 33.39% of the item and to ligation and blank space relocation 64.92% of the items. Additionally, the substitution letters in this condition were from the same Letter group as the substituted letters, so that they differed only in the dots above or below them 16.95% of the time. For instance, the base word ألطمها ?lt^ʕ^mhaa ‘*smack*,’ is converted into the prime أدظمها *ʔdð^ʕ^*mhaa*, where one letter, the ـطـ, is replaced by the same Letter group ظـ, while the second letter لـ is replaced by the different Letter group د. Despite the overall similarity between the two items, it is important to note the new blank space after the second letter of the sequence أد_ظمها, as well as the fact that the letter ظـ ligates to the right in the resulting prime while the letter ـطـ ligates both to the right and left in the base word. In the remaining 83.05% of cases, the substituted letters were from different Letter groups.[Table-anchor tbl3]

Beyond this, the transposed and substituted letters were always the same two root letters in the base word. In half of the stimuli the first and second letters of the root were manipulated, and in the other half the second and third or the second and fourth letters were manipulated. The manipulated letters were never the first or the last letter of the whole word. Finally, none of the roots resulting from letter transposition or letter substitution corresponded to an existing root in the language. Nine different verb word patterns were used with an average type frequency of 4913 (range: 2.4–26265) in the Aralex database.

We also created 60 grapho-tactically legal nonwords by changing one to two consonant letters of an existing Arabic word to be used as targets in the ‘nonword’ response condition. For example, the existing Arabic words انقهرت ʔ*nqhrt* ‘*be conquered*’ and أسعدتا ‘ʔsʕdtaa’ *make happy* are respectively converted into the nonwords انكهزت *ʔnkhzt by changing two consonants and أسعظتا *ʔsʕd^ʕ^*taa* by changing one consonant. These items ranged from 5–8 letters in length (mean: 6.13, SDEV: .57). Each of these nonwords was preceded by one of six types of primes built along the same lines as in the word response (see Table 3). The first condition was an Identity prime (e.g., انكهزت – انكهزت *ʔnkhzt-ʔnkhzt), and the sixth was an all-letter-different baseline nonword prime (e.g., يتخاقم *ytxaaqm). Conditions 2 to 5 constituted the critical manipulation of the experiment by orthogonally crossing Type of Change (letter transposition vs. substitution) with Allography (– Allography vs. + Allography). Specifically, Condition 2, called ‘TL−Allog,’ paired a base nonword target (e.g., انكهزت *ʔnkhzt) with a prime in which two letters were transposed without causing any allographic changes (e.g., انهكزت *ʔnhkzt). Condition 3, TL+Allog, comprised primes in which the transposition of two letters of the base nonword caused allographic changes in the resulting prime. These changes affected the letter form 45.42% of the time, letter ligation 20% of the time, and letter ligation and blank space location 34.58% of the time. In Condition 4, the SL−Allog, two letters of the target nonword were substituted without resulting in any changes either in the form of the residual letters of the base nonword, its ligation pattern or the distribution of blank spaces within it (e.g., انطعزت* - انكهزت* *ʔnt^ʕ^ʕzt- *ʔnkhzt. In this condition, the substituting letters were from the same Letter group as the substituted letters in 3.34% of the items. In Condition 5, SL+Allog, the two letters that were substituted in the nonword target caused allographic changes that pertained to letter form 16.61% of the time, to letter ligation in 12.71% of the cases, and to a combination of letter ligation and blank space location 70.68% of the time. The substituting and the substituted letters were from the same letter Group 4.92% of the time. The transposed and substituted letters were always the same two root letters in the base word, and none of the roots resulting from letter transposition or letter substitution exists in the language. In half of the stimuli the first and second letters of the nonexisting root were manipulated, and in the other half, the second and third letters were manipulated. Five different verb word patterns were used to form this set of stimuli, with an average type frequency of 7499 (range: 4.85–26265) in the Aralex database. Full lists of the stimuli are provided in Appendices A1 and B1.

#### Procedure

Participants were tested in groups of nine at carrel desks in a quiet room. The presentation of the stimuli and recording of response times were controlled by portable laptops using Superlab 5. On each trial, a forward mask consisting of 22 vertical lines (اااااااااااااااااااااا) in a 48-point Arabic font size was presented for 1,000 ms. For cursive Arabic scripts, this mask is preferred to the more commonly used hash mark mask used with stimuli in the Roman alphabet based on the results of several piloting tests, and has been used in previous masked priming studies with Arabic (Boudelaa & Marslen-Wilson, 2004, 2005, 2011, 2015). Next, a prime in 24-point Arabic font was presented for 50 ms, and was then replaced by a target word or nonword in 36 Arabic font size. The target remained on the screen either until the participant’s response or for 2,000 ms, whichever occurred first. Participants were instructed that they would see strings of Arabic letters and that their task was to press the button marked نعم “YES” with their right index finger if the target was a word, and the button marked لا “NO” with their left index finger if the target was a nonword. Participants were instructed to make this decision as rapidly and as accurately as possible. When debriefed at the end of the experiment, none of the subjects reported conscious knowledge of the priming items. Each participant received a different order of trials. The experiment began with 32 practice trials with properties similar to those of the experimental trials followed by the experimental trials. The whole session lasted approximately 10 minutes.

### Results

In this experiment, as well as the next two, the preliminary treatment of the data was as follows: Incorrect responses and response times less than 200 ms or greater than 1800 ms were removed. For the *word response* data, this procedure resulted in the removal of 1.49% of all the data (.98% errors and .50% outliers). For the *nonword response* data, 5.47% of the total data were excluded (2.65% errors and 2.82% outliers). The full raw data set for this experiment and the next two is accessible here: https://osf.io/pmvxk/?show=revision. Mean RTs and error rates for *word* and *nonword* trials are shown in Table 4.[Table-anchor tbl4]

#### Word responses

We analyzed the four critical conditions (i.e., TL−Allog; SL−Allog, TL+Allog, and SL+Allog) using a linear mixed effects modeling approach. We first examined the shape of reaction time (RT) distribution for correct trials requiring the WORD response (a total of 2360 observations for the four conditions under consideration). All RTs were log transformed to best approximate a normal distribution, and to meet the assumption of the linear mixed effects model. The data were then submitted to a linear mixed-effects model using the lme4 package implemented in R 3.2.3 (Bates, Maechler, Bolker, & Walker, 2013). The model included as fixed factors the RTs on the previous trial (PrevRT), along with a full factorial combination of Type of Change (i.e., *Transposed vs. Substituted*), Allography (i.e., *plus allography vs. minus allography*; both of which were deviation-contrast coded), and random intercepts and random slopes for *subjects*, *prime words* and *target words*. All models used for RT latencies and error count measures contained the full random structure as recommended by Barr, Levy, Scheepers, and Tily (2013). If a model containing the full random structure failed to converge, it was systematically pruned by removing interactions between random effects until the model converged. Thus all results reported here are based on successfully converging models.

After removing the RTs of error trials from both the current and previous trials, there were 2325 data points. The degrees of freedom and the *p* values in this experiment and the next two were estimated using Satterthwaite’s approximation as implemented in the lmerTest package (Kuznetsova, Brockhoff, & Christensen, 2013). Neither the main effect of Type of Change, *F*(1, 113.69) = 3.570, *p* = .06, nor that of Allography, *F*(1, 48.62) = .288, *p* = .59, or their interaction was significant, *F*(1, 154.47) = .085, *p* = .77. The only significant effect in this model was that of PrevRT, *F*(1, 2245.76) = 17.61, *p* < .001. We also conducted pairwise post hoc comparisons systematically contrasting TL−Allog with TL+Allog and SL−Allog, as well as the SL+Allog with the SL−Allog and the TL+Allog using Bonferroni-Holm protection levels. The results of these comparisons indicated that performance in the TL−Allog condition did not significantly differ from performance in TL+Allog (*p* = 1.0), SL−Allog (*p* = 1.0), or indeed SL+Allog (*p* = .6). Similarly, the TL+Allog condition did not differ significantly either from the SL−Allog (*p* = .1) or AL+Allog (*p* = 1.0), and finally, the two SL conditions were not significantly different from each other (*p* = 1.0). As a paradigm check, we assessed priming in the Identity condition against the unrelated baseline, the two TL conditions, and the two SL conditions. In each of these comparisons, the identity condition showed significant facilitation (all *p*s < .01), suggesting that the absence of facilitation in our four conditions of interest was not due to lack of statistical power, but was a genuine outcome resulting from the processing dynamics engaged by the primed lexical-decision task in Arabic.

In a second series of analyses, we also evaluated the potential effects of two variables on priming. The first was Form of Allography, which focused on the TL+Allog and the SL+Allog contrast and aimed at assessing the extent to which the three forms of allography (i.e., change in letter form, change in ligation, and change in blank space location within the word) can affect priming. The second, called Letter Group, focused on the SL−Allog and the SL+Allog conditions and sought to determine if priming was modulated by whether the letters substituting each other belonged to the same letter group. Accordingly, we coded the data into four categories on the basis of whether they comprised (a) two substituted letters belonging to the same group and occurring in the same position across prime and target, (b) two substituted letters belonging to the same group, but occurring in different positions, (c) one substituted letter belonging to the same group, and occurring in the same position, and (d) one substituted letter belonging to the same group, but occurring in different positions across prime and target.

The first model revealed no significant effects either for Type of Change, *F*(1, 795.82) = 1.6166, *p* = .203, Form of Allography, *F*(2, 41.30) = 1.7723, *p* = .17, or their interaction, *F*(2, 795.40) = .0354, *p* = .97. The only significant effect in this model was that of the control variable PrevRT, *F*(1, 1108.55) = 18.115, *p* < .001. Similarly, the second model suggested that neither the effects of Allography, *F*(1, 51.36) = .107, *p* = .74, nor those of Letter Group, *F*(3, 82.07) = 1.4374, *p* = .24, or indeed the interaction between them was significant, *F*(2, 95.20) = 1.132, *p* = .33. PrevRT was however, highly significant, *F*(1, 1086.94) = 19.047, *p* < .001.

The error data were analyzed using a logit mixed model (Jaeger, 2008) with the same fixed and random factors as used in the RT models except for PrevRT. The results did not reveal any significant changes in the log odds of the response variable as a function of the predictor Type of Change (*t* = .267, *p* = .79), Allography (*t* = .383, *p* = .70), or their interaction (*t* = .185, *p* = .85). We also ran two further mixed effects models focusing on the possible modulatory effects of Form of Allography in the TL+Allog and the SL+Allog conditions and on those of Letter Group within the two substituted letter conditions. The first model revealed no significant effects for Type of Change (*t* = .285, *p* = .78), Form of Allography (*t* = .292, *p* = .77), or the interaction between them (*t* = −.282, *p* = .77), and neither did the second model, revealing unreliable effects for Allography (*t* = .440, *p* = .66), Letter Group (*t* = −.107, *p* = .92) and their interaction (*t* = .131, *p* = .90).

Given the absence of a significant NHST effect of TL priming in this experiment, we decided to investigate the effect further using Bayes Factors. Not only does a Bayes factor analysis allow us to quantify the relative evidence both for and against the hypothesis that there is an effect of TL, but it also addresses the possibility that the absence of a significant TL effect in NHST might be a consequence of lack of power. If a Bayes Factor analysis gives strong support for the absence of TL priming, this cannot be a result of lack of power. We calculated the Bayes factor using the Bayes factor package (Version 9.12–2, Rouder & Morey, 2013) available in R to compare two mixed effects models that differed in the inclusion of the variable Type of Change, which contrasted letter transposition with letter substitution. Model 1 was the mixed effects model described above, and Model 2 did not include Type of Change. We then used the “compare function” with the default JZS prior to compute the Bayes Factor using Model 1 as the denominator. Our Bayes factor (BF_01_) was 73 ± 4%, thus providing very strong evidence for the null hypothesis that there is no TL priming effect in lexical decision in Arabic.

#### Nonword responses

The nonword RT data, which consisted of 2290 data points after removal of false alarms and outliers, were submitted to the same analyses as the word data, first with Type of Change and Allography as fixed factors, and random intercepts and slopes for *subjects*, *prime words* and *target words*. No significant effects were found for Type of Change, *F*(1, 8.93) = .077, *p* = .78, Allography, *F*(1, 97.37) = .415, *p* = .52, or indeed their two-way interaction, *F*(1, 221.12) = .049, *p* = .82. We did not explore the effects of Form of Allography or Letter Group with the nonword data because the effects of letter transposition and letter substitution were numerically in the wrong direction with the pooled TL conditions averaging 759 ms against a pooled average of 750 ms in the SL conditions.

Turning to the error data for the nonwords, a logit mixed model (Jaeger, 2008) with the same fixed and random factors as used with the word error data revealed no significant effects of Type of Change (*t* = .116, *p* = .91), Allography (*t* = .508, *p* = .61), or their interaction (*t* = 1.930, *p* = .05). A second model focusing only on the two allographic conditions (i.e., TL+Allog and SL+Allog) revealed the effects of Type of Change (*t* = .306, *p* = .76), Type of Allography (*t* = .408, *p* = .68) and their interaction (*t* = −.558, *p* = .58) to be nonsignificant. Another model focused on the two substitution letter conditions (i.e., SL−Allog and SL+Allog) and yielded no effects of Allography (*t* = −.960, *p* = .34), Letter Group (*t* = −.138, *p* = .89), or their interaction (*t* = .146, *p* = .88).

Finally, we sought to quantify the amount of evidence for the presence of orthographic effects in lexical decision by comparing two logit models that differed in the inclusion of the variable Type of Change (i.e., letter transposition vs. letter substitution). Using the “compare function” with the default JZS prior to compute the Bayes factor with the model including Type of Change as the denominator, revealed the Bayes factor (BF_10_) to be 299 ± 7%, suggesting that there was very little evidence for the effects of the variable Type of Change. This corroborates the idea that TL priming is highly unlikely to obtain in lexical decision in Arabic.

### Discussion

This experiment used masked lexical decision priming to determine whether TL priming effects can be observed in Arabic and, if they do, whether they are modulated by allographic variation. The results clearly show that TL priming effects do not obtain in this language. This outcome replicates the pattern observed consistently with Hebrew, another Semitic language with the Semitic morphology based on consonantal roots (Velan & Frost, 2009, 2011) and extends the results originally reported by Perea et al. (2010) for Arabic word primes to nonword primes. Furthermore, our post hoc analyses suggest that the different types of allography (i.e., change in letter form, letter ligation, or blank space location) behave in the same way, consistent with previous reports in the literature (Perea, Abu Mallouh, Mohammed, Khalifa, & Carreiras, 2016). Similarly, the fact that priming was not modulated by whether or not the manipulated letters came from the same Letter Group suggests that, at least in lexical decision, individual letters and allographs are treated as wholes and not analyzed into a letter shape of some sort plus a number of superposed dots. We do acknowledge though that these two outcomes are based on post hoc analyses of a subset of the data and should, therefore, be taken with due caution.

More generally, our results can be accommodated within the *noisy position* Bayesian Reader (Norris & Kinoshita, 2012a) as follows. According to this view, word recognition consists of accumulating evidence from the visual input via noisy perceptual sampling. Within this framework a *yes* response in the lexical-decision task is made when there is enough evidence to decide that the input is more likely to be a word than a nonword, whereas a *no* response is made when there is enough evidence to determine that the input is unlikely to have been generated by a word (Norris, 2006; Norris & Kinoshita, 2008). Because lexical decision is standardly thought to trigger lexical processes, this view implies that the amount of evidence needed to make a decision will be modulated by the structural and distributional characteristics of the lexical space. In the present case, we independently know that the lexical status of a string of letters in Arabic is primarily determined by whether its root exists or not (Boudelaa, 2014; Boudelaa & Marslen-Wilson, 2015). We also know that the lexical space in which roots are organized is densely populated such that transposing two letters in any of the commonly used 4858 Arabic triliteral and quadriliteral roots results in an existing root 54% of the time.^4^ As an example, consider the Arabic root (e.g., {rtb} *tidying up*), which consists of three consonants arranged in a specific order. If the order is changed, completely different roots are obtained (e.g., {rbt} *patting*, {trb} *sand*, {tbr} *gold*, {brt} *ax*, {btr} *cutting off*), suggesting that the lexical space in Arabic is dense compared with that of a language like English where the rearrangement of letters within a word (e.g., *right, can, moot*) typically yields nonexisting word (e.g., *rihgt, nac, toom*).

It is well established in the masked priming literature with European languages written in the Roman alphabet that the “neighborhood density constraint” operates: Specifically, in lexical decision, orthographic priming effects by (substituted-letter/SL) nonword neighbor primes (e.g., bamp-CAMP; bontrast-CONTRAST) are weak or absent when the prime and target are drawn from high-density neighborhoods (Forster, Davis, Schoknecht, & Carter, 1987). In Semitic languages like Arabic, the lexical space is structured around the triconsonantal root, and it is densely populated by TL (as well as SL) neighbor roots. In lexical decision then, TL priming effects are difficult to observe in Semitic languages, as has been shown repeatedly by Frost and colleagues (e.g., Velan & Frost, 2009, 2011) in Hebrew, and in Arabic by Perea et al. (2010), and in the present experiment. In this view, the cross-language contrast in the finding of TL priming effects in lexical decision is due to the density of lexical space, and the structure of the orthographic representations that comprise the lexical space. In English and other European languages written in the Roman alphabet the orthographic representations that constitute the lexical space are a linear sequence of abstract letter identities. This lexical space is sparsely populated by TL neighbors. In contrast, the Semitic morphological structure is nonlinear, with the word pattern interleaved with the triconsonantal root. The lexical space is organized around the triconsonantal root, and is densely populated with TL neighbors.

A further implication of *the Bayesian Reader* view of masked priming is that if we were to use a task that does not engage the lexical access process, such as the same–different match, the otherwise pervasive effects of the Semitic morphological structure should have little impact. As noted, in this task, the input is not matched against the whole lexicon, but only against the single referent item. Because the priming effects should now reflect the matching process against the prelexical orthographic representation consisting of abstract letter identities we should see the emergence of TL-priming which should then be modulated by allographic variation. This is the goal of Experiment 2.

## Experiment 2

This experiment used the same–different match task to test whether the TL priming effect is modulated by allographic variation in Arabic words. In this task, participants are first presented with a referent and are asked to decide whether a subsequently presented target is the same as or different from, the referent. The masked priming procedure is otherwise identical to that used in the lexical-decision task with a forward mask consisting, in this case, of 22 vertical lines, followed a briefly presented prime that is in turn backward-masked by the target so that participants are unaware of the identity of the prime. According to the *noisy channel Bayesian Reader* view (Norris, 2006; Norris & Kinoshita, 2012a), TL priming effects in this task arise because in the brief time the prime is available for processing, the spatial position of adjacent letters, and hence their relative order, is uncertain. As noted, allographic variation in Arabic is position-dependent, with some letters changing shape depending on their position within a word. Friedmann and Haddad-Hanna’s (2012) findings suggest that Arabic readers suffering from Letter Position Dyslexia use allographic variation as a cue to reduce the uncertainty in letter position, as the letter form can provide a cue regarding the letter’s position. A prediction that follows from this is that allographic variation would modulate the TL priming effect such that the effect would be reduced when allographic letters are transposed compared with when nonallographic letters are transposed. In addition, our letter transposition manipulation always involved root letters. Hence the experiment also served as a replication of Perea et al. (2011, Experiment 1), testing their claim that the same–different task is sensitive to morphology and hence TL priming effect would be absent when the transposition involves root letters.

### Method

#### Participants

Sixty-seven female volunteer students from the United Arab Emirates University participated in the experiment. They were aged 20–24 and were native speakers of dialectal Emirati Arabic. They all used Modern Standard Arabic on a daily basis, and had normal or corrected-to-normal vision. The sample size was guided by, and is much greater than, previous studies of TL priming same–different experiments in Arabic (Perea et al., 2011, 20 participants).

#### Stimuli and design

The stimuli and design for the *same* response trials were identical to those used in Experiment 1 for the yes-response except for the use of the target items as a reference for *the same* response condition. Table 5 displays sample stimuli used in this experiment.[Table-anchor tbl5]

For the *different* response condition, we matched another 60 words to those used in the same response condition and used them as targets. These targets ranged in length between five and eight letters (mean 6.35 letters), with an average frequency of 16.86 (range: .03–237.42). They were based on six different verb word patterns with an average frequency of 4193 (range: 2.41–26265). The construction of the transposed and substituted letter with and without allography conditions as well as the construction of the unrelated and identity primes was identical to that of the trials in the *same* response condition. For the TL+Allog condition, the allographic changes consisted of letter form changes in 42.54% of the items, of ligation changes in changes 22.24% of the time, and of a combination of ligation changes and blank space relocation in 35.22% of the items. For SL+Allog, 10.75% of the changes were letter form changes, and another 10.75% were ligation changes, while the remaining 78.51% were a combination of ligation changes and blank space relocation. Additionally, the substituting and the substituted letters were from the same letter Group 9.40% of the time, whereas for the SL−Allog condition the manipulated letters came from the same letter Group 21.34% of the time. The *different* response reference items consisted of a further 60 words with an average length of 6.25 letters (range: 4–8) and an average frequency of 22.18 (range: .03–244.73). To maximize the difference between the reference words and their associated targets, the references were built using as many verb word patterns as possible—more specifically, using 12 of the 15 patterns existing in the language. Their average frequency was 4592 (range: 2.14–26265). A series of paired *t* tests revealed no differences between the experimental items in the various conditions in terms of their distributional characteristics (all *p*s > .5).

Six experimental lists were constructed so that each target appeared once in each set, but each time in a different priming condition. Different groups of participants were randomly assigned to each list. The full list of stimuli is in Appendixes A1 and A2.

#### Procedure

The general procedure was identical to that in Experiment 1 with the exception that each trial began with the presentation of a reference stimulus in 36-point Arabic font above a forward mask consisting of 22 vertical lines (ااااااااااااااااااااا) in a 48-point Arabic font size for 1,000 ms. Next, the reference disappeared, and the forward mask was replaced by a prime in 24-point Times New Roman Arabic font presented for 50 ms. Then, the prime was replaced by the target word in 36 Times New Roman Arabic font size. The target remained on the screen either until the participant’s response or for 2,000 ms, whichever occurred sooner. Participants were instructed that they would see strings of Arabic letters and that their task was to press the button marked نعم “YES” with their right index finger if the reference and target were the same, and the button marked لا “NO” with their left index finger if the reference and target were different. Participants were instructed to make this decision as rapidly and as accurately as possible. When debriefed at the end of the experiment, none of the subjects reported conscious knowledge of the priming items. Each participant received a different order of trials. The experiment began with 32 practice trials with properties similar to those of experimental trials followed by experimental trials. The whole session lasted approximately 12 min.

### Results

The analysis of RTs and errors followed the same procedure as Experiment 1 and revealed an overall error rate of 5.17% for the *same response* data (5.14% errors and .024% outliers). For the *different response* data, 4.70% of the overall data were excluded (4.67% errors and .024% outliers). Mean RTs and error rates for the *Same* and *Different* trials are shown in Table 6.[Table-anchor tbl6]

#### Same responses

As in the previous experiment, we analyzed the four critical conditions (i.e., TL−Allog; SL−Allog, TL+Allog, and SL+Allog) using a linear mixed effects modeling approach. We examined the shape of the RT distribution of the correct trials requiring the SAME response (a total of 2680 observations for the four conditions under consideration), and log-transformed them to best approximate a normal distribution, and to meet the assumption of the linear mixed effects model. We then fitted the full random structure linear mixed effects model with RTs on the previous trial (PrevRT) as fixed factors and a full factorial combination of Type of Change (i.e., *Transposed vs. Substituted*), and Allography (i.e., *plus allography vs. minus allography*; both of which were deviation-contrast coded) and random intercepts and slopes for *subjects*, *prime words* and *target words*. If the full random structure model failed to converge, we trimmed it until it did. After removing RTs on error trials from both the current and previous trials, there were 2573 data points, with 67 subjects and 40 targets.

The main effect of Type of Change was significant, *F*(1, 76.11) = 59.008, *p* < .001, indicating a significant TL priming effect overall. Similarly, the main effect of Allography was also significant, *F*(1, 172.87) = 13.607, *p* < .001], as was that of PrevRT, *F*(1, 2501.68) = 46.526, *p* < .001. More importantly, the interaction between Type of Change and Allography was also significant, *F*(1, 173.95) = 4.260, *p* < .04, as indicated by the reduced TL priming effects in the +Allography condition shown in Table 6. Pairwise comparisons using Bonferroni-Holm tests yielded a statistically reliable difference between TL−Allog and SL−Allog (*p* < .001) and the TL+Allog and SL−Allog (*p* < .001).

We then ran two models. The first focused on the TL+Allog and the SL+Allog conditions to assess whether Form of Allography (i.e., letter shape change, ligation change and blank location change) modulated response times differently. The results revealed that Type of Change (i.e., letter transposition vs. letter substitution) had a significant effect, *F*(1, 56.50) = 25.159, *p* < .001, whereas Form of Allography, *F*(2, 53.07) = .994, *p* < .38, and the two-way interaction Type of Change by Form of Allography, *F*(2, 68.64) = .739, *p* = .48, did not. The control variable PrevRT had a significant effect, *F*(1, 1243.62) = 47.126, *p* < .001. The second model focused on the two SL conditions with a view to determining whether response latencies were differentially modulated by Letter Group, that is by whether the substituted letters belonged to the same letter group or not. The results indicated that while the effects of Allography, *F*(1, 5.55) = 1.231, *p* = .27, and the interaction between Allography and Letter Group, *F*(2, 111.04) = 1.277, *p* = .28, were not significant, those of Letter Group, *F*(3, 69.98) = 4.493, *p* < .006, and PrevRT, *F*(1, 1231.99) = 42.969, *p* < .001, were reliable.

Turning to the errors in the same response data, we used a logit mixed model approach as in Experiment 1, with the same fixed and random factors as used in the RT model except for PrevRT. This analysis revealed no significant effects of Type of Change (*t* = −1.475, *p* = .14), Allography (*t* = −.415, *p* = .69), or the interaction between them (*t* = 1.238, *p* = .22). We also carried out two sets of analyses with the same structure as above except that the first focused solely on the TL+Allog and the SL+Allog conditions, and included only Type of Change and Form of Allography as fixed factors, while the second focused on the two SL conditions and included only Allography and Letter Group as fixed factors. For the first model, the results showed that neither Type of Change (*t* = .417, *p* = .68), Form of Allography (*t* = −.123, *p* = .90), nor the interaction between them (*t* = 0.248, *p* = .80) was reliable. Analogously, the second model also revealed no significant effects for Allography (*t* = −1.283, *p* = .20), Letter Group (*t* = −1.182, *p* = .28), or their two-way interaction (*t* = 1.448, *p* = .15).

Finally, we computed the Bayes factor to quantify the amount of evidence for the two-way interaction Type of Change × Allography by comparing two mixed-effects models that differed in the inclusion of this interaction term. Model 1 was the mixed effects model described above, while Model 2 did not include the two-way interaction. We then used the “compare” function with the default JZS prior to calculate the Bayes factor with Model 2 as the denominator. The Bayes factor was 21 ± 5.24%, suggesting very strong evidence for the interaction—that is for the hypothesis that the magnitude of priming was modulated differently by allography.

#### Different responses

The same statistical analyses as above were applied to the Different trials, and consistent with the literature (Norris & Kinoshita, 2008), revealed no effects either for Type of Change, *F*(1, 173.77) = .056, *p* = .81, Alllography, *F*(1, 178.39) = 2.073, *p* = .15, or their interaction, *F*(1, 18.17) = .330, *p* = .57. The analysis of the potential effects of Form of Allography in the context of the TL+Allog and the SL+Allog conditions revealed no significant effects for Type of Change, *F*(1, 59.76) = 0.211, *p* < .65, Form of Allography, *F*(2, 78.96) = 1.473, *p* = .24, or the interaction between them, *F*(2, 107.75) = 2.257, *p* = .11. The only significant effect was that of the control variable PrevRT, *F*(1, 1218.12) = 40.809, *p* < .001. Similarly the analysis focusing on the two substituted letter conditions revealed no significant effects for Allography, *F*(1, 56.34) = 1.7814, *p* = .19, Letter Group, *F*(2, 115.40) = 0.3204, *p* = .726, or their interaction, *F*(2, 120.58) = 2.5943, *p* = .08; only the effects of PrevRT were significant, *F*(1, 1263.89) = 20.7096, *p* < .001.

The error responses to Different trials were also submitted to the same logit model as those of the Same responses. The effects of Type of Change (*t* = −1.475, *p* = .14), Allography (*t* = −.415, *p* = .69), and their interaction (*t* = 1.238, *p* = .22) were not significant. Further post hoc analyses of the two allography conditions (i.e., TL+Allog and SL+Allog) yielded no significant effects of Type of Change (*t* = −0.341, *p* = .74), Form of Allography (*t* = −1.192, *p* = .23), or their interaction (*t* = 1.285, *p* = .20). Similar analyses of the two SL conditions indicated that the effects of Allography (*t* = −1.161, *p* = .25), Letter Group (*t* = −1.544, *p* = .12) and their interaction (*t* = 1.257, *p* = .21) were not significant.

### Discussion

The results of this experiment are clear: In Arabic, the same–different matching task reveals a robust TL priming effect that is modulated by allographic variation. This finding contrasts with the extant literature on Arabic word recognition in two respects. First, the finding of TL priming effect in the same–different task when the transposed letters are root letters is at odds with the absence of such an effect reported by Perea et al. (2011). The present finding, however, replicates the effects reported by Kinoshita et al. (2012) for Hebrew, another Semitic language, but with a much more limited position-dependent allography. Surprisingly our results also suggest that the different forms of allography (i.e., form change, ligation change, and blank space location) do not exert differential modulatory effects on priming. There was no significant interaction between Type of Change and Form of Allography for either the latency or error data, and there was an absence of an effect for Form of Allography. However, this finding needs to be interpreted with caution because it is based on post hoc analyses with unmatched numbers of observations. For instance, 42.5% of the allographic changes pertained to changes in Letter Form in the TL+Allog condition but only 1.69% were Letter Form changes in the SL+Allog condition. With respect to the effects of Letter Group, our post hoc analyses suggest however that this variable can modulate the effects of letter substitution, with letters substituted from the same letter group yielding more priming than those substituted from a different group. Although this outcome makes intuitive sense, it has to be interpreted cautiously for a number of reasons. First, our experiment was not specifically designed to evaluate the effects of Letter Group. Second, the effects of this variable were not significant in either Experiment 1 or Experiment 2. Finally, these effects are inconsistent with a recent report by Perea et al. (2016), who showed that priming effects in Arabic did not vary as function of Letter group membership: A target like صحفية *s*^ʕ^*ℏfyyt* ‘*journalist*’ has comparable latencies when preceded by an SL prime from the same Letter group (e.g., صخفية* *s*^ʕ^*xfyt*) or a different Letter group (e.g., صكفية* *s*^ʕ^*kfyt*), suggesting that information about the dots is rapidly uptaken by the lexical processing system. The foregoing discussion illustrates the elusive nature of letter group effects and calls for further work to elucidate the true role of this variable.

The TL priming effect in the same–different task is interpreted as having its origin in the noisy perception of letter order, and is not assumed to be a consequence of the structure of the language (cf. Gómez et al., 2008; Kinoshita & Norris, 2009). In the same–different task, which does not require lexical access, TL priming effects are unaffected by morphological structure (Kinoshita et al., 2012). This contrasts with tasks such as lexical decision where morphological structure does influence processing at an early stage, and where integration of evidence about roots and word patterns is crucial to making the decision. This is well documented in the work of Boudelaa and Marslen-Wilson (2005), who reported strong root priming effects at an SOA of 32 ms in Arabic, and the work of Friedmann and Gvion (2001), who described two Hebrew patients with peripheral dyslexia whose migration errors were significantly modulated by the morphological structure of the word. Our point here is that word recognition is a dynamic process during which evidence is accumulated to implement a task-driven decision. In the same–different match task, the decision concerns whether the target item matches a single reference item. Masked priming in this task is insensitive to the morphological structure (Duñabeitia et al., 2011), and (in the brief period of time that the prime is available) the prime is coded as a linear sequence of abstract letter identities. In contrast, in lexical decision, the decision is heavily based on whether the target item matches (any) item(s) in the lexicon, which, in Semitic languages, is organized in terms of nonconcatenative roots + word patterns. Thus during this task, the linguistic processor has to constantly gather evidence about roots and word patterns, and evaluate (a) whether these two components exist in the lexicon, and (b) whether their combination is licensed—that is, a real word and not a morphologically structured pseudoword. It is the ubiquitous morphological structure that characterizes Arabic words and the TL and SL nonwords as used here that determines the organization of the lexicon, triggers morphemic parsing in any task requiring lexical access, and ultimately imposes higher decision criteria during lexical decision.

The present finding of robust TL priming effects in Arabic with the letter transposition manipulation involving the root letters reinforces this interpretation. It argues against Perea et al.’s (2011) interpretation that Semitic morphology modulates TL priming effects in the same–different task. Given the many differences between our study and Perea et al.’s, particularly their failure to control important properties of the stimuli including the position-dependent allography, it is hard to pinpoint why TL priming was absent in their study. One plausible explanation for this discrepancy between the two studies however is that we used more than twice the number of participants they did, and it may well be that their study lacked the necessary power to detect significant TL priming.

Second, and more importantly, this is the first demonstration that allographic variation modulates masked priming effects. On the surface, this finding seems to contrast sharply with those reported by Carreiras, Perea, and Abu Mallouh described earlier. On a closer look, however, there is no real discrepancy between our results and theirs. Recall that Carreiras et al. (2012, 2013) used single letters and found that the visual similarity of allographs did not modulate the size of identity priming effects. Presented as a single letter, allographic variation in Arabic has nothing to say about letter position and hence it is not surprising that the visual similarity manipulation functioned just like the visual similarity of the allographs in the Roman alphabet (e.g., *a/A vs. c/C*) reported by Kinoshita and Kaplan (2008). In a different study using the lexical-decision task, Perea et al. (2013) did use word stimuli and manipulated the visual similarity of allographs substituted in the prime, and still found that it did not modulate the size of priming. As also noted, however, the critical allograph similarity manipulation concerned nonroot letters and the task used by Perea et al. was lexical decision, so the absence of the allograph similarity effect can be explained in terms of the view that lexical processing in Arabic is tuned to pick up root consonants (Boudelaa & Marslen-Wilson, 2005, 2013, 2015).

## Experiment 3

Experiment 2 used stimuli that consisted of words as referents and targets to show that TL-priming effects were modulated by allographic changes caused by letter transposition. Experiment 3 strengthens this case by showing that priming in the same–different matching task obtains even with nonword stimuli thus reinforcing the claim that the position-dependent allography impacts prelexical orthographic representations.

### Method

#### Participants

Sixty-two female volunteer students from the same population as in Experiments 1 and 2 took part in this experiment. They were 20 to 24 years of age and were native speakers of dialectal Emirati Arabic who used Modern Standard Arabic on a daily basis, and had normal or corrected-to-normal vision. The choice of this sample size was informed by common practice in the field.

#### Stimuli and design

A 60 nonword set was used in the *same response*. It was built by changing one to two consonant letters in an existing word to create an ortho-tactically legal nonword ranging between five and eight letters in length (mean: 6.13). They were formed using five different word patterns with an average frequency of 7499.86 (range: 4.85–26265). The construction of the transposed and substituted letter with and without allography conditions as well as the construction of the unrelated and identity primes was identical to that of the trials in the *same response* condition of Experiment 2. For the TL+Allog condition, the allographic changes were letter form changes 55.32% of the time and ligation and blank space location changes 44.68% of the time. For the SL+Allog condition these changes were letter form changes 1.96% of the time and Ligation and blank space location changes 98.39% of the time. Additionally, the substituted letters in this condition did not belong to the same letter group. The same holds for the SL−Allog, where none of the substituted letters came from the same letter group.

The *different response* set consisted of another 60 nonwords also built by changing one to two consonants of an existing word to form a legal orthographic string. They ranged in length between 5 and 8 letters (mean 6.25 letters). These items were based on five different verb word patterns with an average frequency of 6652 (range: 4.85–26265). The construction of the transposed and substituted letter with and without allography condition as well as the construction of the unrelated and identity primes was identical to that of the trials in the *same response* condition. The *different referents* were made up of a further 60 nonwords with an average length of 6.22 letters (range: 5–8), also built by changing one or two letters of an existing word. Seven different verb word patterns were used with these items with an average frequency of 1573 (range: 4.85–26265). Where the form of allography is concerned, the changes in the TL+Allog condition consisted of letter form changes 50.16% of the time and ligation and blank space relocation 49.84% of the time. In the SL+Allog, the changes were letter form changes 1.61% of the time, ligation changes 82.58% of the time, and ligation and blank space relocation 15.81% of the time. Further, in this condition, the proportion of the substituted letters belonging to the same letter group was 1.61%, whereas for the SL−Allog, this proportion was 21.61%. Six experimental lists were constructed so that each target appeared once in each set, but each time in a different priming condition as illustrated in Table 7. Different groups of participants were randomly assigned to each list.[Table-anchor tbl7]

#### Procedure

This was identical to Experiment 2.

### Results

Applying the same pruning procedure as in the previous two experiments resulted in the exclusion of 7.15% of the data points (7.07% errors and .08% outliers) from the *same response* data, and 5.99% for the *different response* data (5.91% errors and .08% outliers). This left 2331 data observations in the four conditions of interest. We fitted the full random structure model with Type of Change (TL vs. SL), Allography (+Allog vs. −Allog), their interaction, previous trial RT (PrevRT) as fixed factors, and Subjects, Primes, and Targets, as random factors each with a random intercept and random slope. When the full structure model did not converge, we pruned it by removing interactions between random factors until it did, and the results we report are all of models that converged. Mean response latencies, magnitude of priming and error rates are presented in Table 8 for the *same* and *different response* trials.[Table-anchor tbl8]

#### Same responses

The latency data analysis revealed the main effect of Type of Change to be significant, *F*(1, 228.63) = 23.582, *p* < .001. Likewise, the main effect of Allography was significant, *F*(1, 71.91) = 20.177, *p* < .001, and most importantly, the two-way interaction Type of Change by Allography was also significant, *F*(1, 199.58) = 10.407, *p* < .001. Pairwise comparisons contrasting TL−Allog with SL−Allog and TL+Allog with SL+Allog using Bonferroni-Holm protection levels, revealed that while TL−Allog was significantly different from SL−Allog [*p* < .001], the two +Allog conditions did not differ significantly from each other (*p* = .65). Further post hoc analyses focusing on the two allographic conditions (i.e., TL+Allog and SL+Allog) showed the effects of Type of Change (i.e., TL vs. SL) to be nonsignificant, *F*(1, 143.22) = 1.050, *p* = .31, and neither were the effects of Form of Allography, *F*(2, 237.44) = 0.699, *p* = .50, nor those of their interaction, *F*(2, 94.12) = 0.296, *p* = .74. In this model, the only significant effect was PrevRT, *F*(1, 1108.83) = 7.4370, *p* < .006. Focusing on the effects of Letter Group within the two-letter substitution conditions revealed no reliable effects for Allography, *F*(1, 43.12) = 1.232, *p* = .27, or Letter Group, *F*(1, 39.58) = 1.148, *p* = .29. There was however a reliable effect of PrevRT, *F*(1, 1055.60) = 3.8150, *p* < .05.

The accuracy data were submitted to a logit mixed model with the same fixed factors as before (excluding previous trial RT). The effects of Type of Change were nearly significant (*t* = −1.911, *p* < .06), while those of Allography (*t* = −0.698, *p* = .49) and the interaction between Type of Change and Allography (*t* = 1.326, *p* = .18) were not. Pairwise post hoc Bonferroni-Holm corrected analyses revealed there to be no difference between any two combinations of the four condition in terms of error rates. We also evaluated the effects of Form of Allography in the context of the TL+Allog and the SL+Allog conditions and found that the effects of Type of Change (*t* = −0.698, *p* = .49), Form of Allography (*t* = 1.515, *p* = .130), and the interaction between them (*t* = −0.505, *p* = .61) were not significant.

Finally, we calculated the Bayes factor for the interaction by comparing our main model, which included the interaction Type of Change × Allography with a model that did not include this interaction as the denominator. The results of this analysis suggest that there was evidence for this interaction with odds equaling 7.96 ± 2.37%. This is consistent with the results of Experiment 2 and suggests that the data provide reliable evidence for the hypothesis that Allography modulates the amount of TL priming effects in the same–different matching task in Arabic.

#### Different responses

Data from the *different response* trials were analyzed in the same way as the *same responses*. Consistent with the literature (Norris & Kinoshita, 2008), and with Experiment 2, the results revealed no significant effects of Type of Change, *F*(1, 85.59) = 0.487, *p* = .49, Allography, *F*(1, 76.81) = 1.066, *p* = .31, or their interaction, *F*(1, 240.22) = 0.768, *p* = .38. The control variable PrevRT was significant, *F*(1, 2204.52) = 63.271, *p* < .001. Pairwise comparisons between the four conditions (i.e., TL−Allog, TL+Allog, SL−Allog and SL+Allog) using the Bonferroni-Holm correction revealed no significant differences whatsoever (all *p*s ≥ .1).

A comparison of the two allography conditions (i.e., TL+Allog and the SL+Allog) to determine whether the different manifestations of allography modulated response time revealed no effects either of Type of Change, *F*(1, 269.20) = 1.897, *p* = .17, Form of Allography, *F*(3, 116.99) = 0.238, *p* = .87, or their interaction, *F*(2, 115.10) = 0.253, *p* = .78. The only significant effect to be found here was PrevRT, *F*(1, 1116.10) = 21.613, *p* < .001.

Error responses to Different trials were also analyzed using the same logit approach used with the *same response* trials. The effects of Type of Change (*t* = −1.940, *p* = .05), Allography (*t* = −0.428, *p* = .67), and their interaction (*t* = 1.472, *p* = .14) were not significant. Similar results emerged from the analysis of the two allography conditions with Type of Change (*t* = −1.775, *p* = .08), Form of Allography (*t* = −0.980, *p* = .33), and their two-way interaction all failing to show any significant effects (*t* = 0.901, *p* = .37). Similarly, the analysis of the two transposition conditions (i.e., SL−Allog and SL+Allog) revealed no significant effects for Allography (*t* = −1.102, *p* = .27) or Letter group (*t* = −0.815, *p* = .42).^5^

### Discussion

Kinoshita and Norris (2009) suggested that the same–difference matching task taps prelexical representations. If this is the case in Arabic, then the TL priming effects observed in Experiment 2 should also be found in Experiment 3. The results clearly indicate that TL priming effects in nonwords are similar to those observed with words and are significantly modulated by allographic variation, in the sense that the critical two-way interaction between Type of Change and Allography proved to be significant in both Experiments 2 and 3. Our post hoc analyses also suggest that Form of Allography (i.e., change in letter from, change in ligation, or blank space location) are treated as functionally equivalent in terms of changing the overall appearance of the word. Transposed and substituted letter words are not perceived as more similar or more different depending on whether the experimental manipulation caused changes in letter form, letter ligation, or blank space location. These analyses further suggest that the variable Letter Group requires further investigation to determine the extent to which it may or may not modulate the effects of letter substitution.

The main conclusion one can draw from this experiment is that masked priming in the same–different task does not operate on representations higher than letters in Arabic, as claimed by Perea et al. (2011). One reason for the discrepancy between the present results and Perea et al.’s may be, as we suggested above, that they did not control for allographic variation. Another reason may be that they sometimes manipulated a *matres lectionis* letter which can be read as a vowel or consonant depending on its position in the word. The results of a recent study by Kinoshita et al. (2012) using the same–different matching task with Hebrew words and nonwords are in keeping with the outcomes of this experiment. Both that study and the present experiments indicate that provided that the influence of position-dependent allography is controlled, even when the letter transposition manipulation is applied to the root letters, TL priming effects emerge in the same–different task thus indicating that this task operates on prelexical representations.

## Combined Analyses of Experiments 1 and 2

In this section, we report an analysis of the data from the four conditions of interest (i.e., TL−Allog, SL−Allog, TL+Allog, and SL+Allog) using the *word response* data of Experiment 1 and the *same response* data of Experiment 2 to better understand the change in the pattern of priming across the two tasks. To perform this between-experiment comparison, response times for each participant were transformed into z-scores (i.e., RTs for target words were expressed as a number of standard deviations greater or less than each participant’s mean) to remove differences between participants in the speed or variability of their responses (Davis, Marslen-Wilson, & Gaskell, 2002; Zwitserlood, 1989). We took the same approach as before, fitting the full structure model and pruning as necessary until the model converged. The main effects of Type of Change, *F*(1, 157.4) = 32.413, *p* < .001, and Allography *F*(1, 245.7) = 7.951, *p* < .005, were significant. Apart from the two-way interaction Type of Change by Task, *F*(1, 157.1) = 10.851, *p* < .001, neither the two-way interaction Type of Change by Allography, *F*(1, 146.0) = .736, *p* = .392, nor Allography by Task, *F*(1, 346.1) = .704, *p* = .40, nor the three-way interaction Type of Change × Allography × Task, *F*(1, 234.7) = 1.581, *p* = .21, was significant. The significant interaction between Type of Change and Task suggests that the effects of allographic variations are modulated by task demands. Previous experimental research corroborates this and suggests that the masked priming lexical-decision task in Arabic engages lexical access processes which project orthographic input onto internal representations that are defined particularly in terms of root morphemes (Boudelaa & Marslen-Wilson, 2005, 2015; Gwilliams & Marantz, 2015; Prunet, Béland, & Idrissi, 2000). This morphological configuration of the Arabic lexical space brings new processing demands to bear on the implementation of the lexical-decision task in the context of Arabic: It has to be implemented on the basis of lexical information that corresponds to the root. Any manipulation that affects the root identity either by transposing or substituting its component letters as we did here will result in a new root that will not be considered by the language processor to be any more similar to the base root than any other random root. For instance, the TL root {tkb} and the substituted letter root {xjb} will not be any closer in lexical space to the base root {ktb} than the random root {hnf}. This leaves little room for TL-priming effects to emerge in Arabic. In contrast, the same–different match task operates on prelexical representations and does not engage lexical access processes hence manipulations that alter the root letters are not detrimental to TL priming effects since performance on the task is not contingent on morphological structure.

## General Discussion

The three experiments reported here provide new evidence that addresses a number of important questions in visual word recognition; in particular, whether TL priming effects in Arabic are modulated by position-dependent letter shape change (allography) and whether different processing dynamics are engaged by lexical decision and the same–different match task. In what follows we first discuss these issues in light of the new evidence our results provide, then we consider the implications of these results for current models of letter coding.

### TL Priming Effects and Allography

The purpose of this research was to determine whether allographic variation plays a role in the orthographic processing of Arabic and whether this role is modulated by task demands. Unlike the Roman alphabet (uppercase and lowercase letters) used in European languages, allographic variation in Arabic is position-dependent. Using a masked priming lexical-decision task (Experiment 1) our findings suggest that when the task engages lexical access processes, the standard TL-priming effects routinely observed in Indo-European languages are not found, nor are they modulated by the change in shape of the letter as it is transposed or substituted. In contrast, Experiments 2 and 3 using the same different match task with words and nonword, respectively, clearly show that robust TL priming effects are present in this task, and that allographic variation modulates TL priming. Priming is reduced for letters that change shape (i.e., involve allographic variation). This result is significant, not only because it is the first such finding in the literature, but also because it shows how the allographic variation in Arabic impacts on orthographic processing. As discussed earlier, Friedmann and Haddad-Hanna (2012) reported that letter migration errors in reading aloud in Arabic LPD (letter position dyslexia) patients are reduced for words in which letter transposition or letter substitution causes allographic changes in their overall shape. The authors attributed this finding to the position-dependent allography unique to Arabic. Our data indicate that Friedmann and Haddad-Hanna’s (2012) results are not a product of an immature reading process specific to individuals with dyslexia relying more on “global, visual elements rather than on abstract letter representations” akin to “visual, logographic level of reading in the initial steps of learning to read” as suggested by Perea et al. (2013, p. 571). Rather, their results, like ours, reflect the adaptive use of linguistic knowledge to counter perceptual uncertainty.

Our results are also consistent with the Uyghur data we mentioned above (Yakup et al., 2014, 2015). These authors acknowledged the role of position-dependent allography in letter position coding in skilled readers based on the finding that reading of “jumbled words” (words with internal letters rearranged) was modulated by position-dependent allography. Yakup et al. (2015) maintained that such effects would be difficult to demonstrate in Arabic even though it uses the same script as Uyghur because of its Semitic morphology and the predominantly consonantal system. Our findings in Experiment 2 and 3 on the modulation of TL priming by position-dependent allography in skilled readers of Arabic clearly shows that these are not the limiting factors.

In most languages using a Roman script, and with the exception of the first letter in a sentence or the first letter in a proper noun, allographic variation does not convey any useful information about either word identity or letter order. Consistent with this, readers appear to rely entirely on a level of letter representation that abstracts over these details. In contrast, allographic variation in Arabic conveys important information about the order of letters within a word and hence the identity of the word and, as shown by the modulation of TL priming by allography, readers successfully take advantage of that information to reduce positional uncertainty. Allographic information must necessarily be associated with letter-identity information. Therefore, Arabic readers must have access to a representation that encodes order, identity, and allographic information simultaneously. Note that this result should not be taken to imply that there is not a level of abstract letter representation in Arabic. The final level of representation driving lexical access must abstract over irrelevant visual details of the script such as variations in font or size. The critical message from the present study is that readers of Arabic are able to use information about the allographic form of letters to constrain letter order which, early on in processing, is ambiguous because of noise in the perception of letter order.

Finally, the current set of experiments provides interesting suggestions about two issues. The first is that the different manifestations of allography (i.e., change in letter form, change in letter ligation and change in blank space location with the word) appear to be equally effective as cues to letter order in the same–different task in Arabic. This finding suggests that the overall shape of the Arabic word might contribute information to constrain the visual processing of the letter string. A second suggestion relates to the effects of letter substitution which do not seem to be modulated by letter group. Specifically, SL primes in which the replaced letter kept the same basic shape as the original letter (e.g., يشغدون* - يسعدون *yʃɣduun- ysʕduun* ‘*be happy*’) did not prove to be more effective at facilitating the target than substituted letter primes in which the replaced letter was visually different (e.g., يهضرون* - يسترون *yhd*^ʕ^*ruun-ystruun* ‘*cover*’). This suggests that information about diacritical marks plays a critical role in defining letter identity and that the diacritics are processed as an integral part of the letter. At a more general level, this finding sheds new light onto the possible detailed specifications of the type of letter representation that needs to be mapped onto an ‘abstract letter level’ in Arabic (cf., Perea et al., 2016 and Wiley, Wilson, & Rapp, 2016).

### Lexical Decision Versus Same–Different Match Task

In line with Norris and Kinoshita’s (2012a) noisy channel model, we view the process of visual word recognition as a special case of general object recognition, and that letter position coding depends on the same processes as other forms of object position coding. Because perception is noisy, both the letter identity information and letter position information are initially ambiguous, but become more certain as more perceptual information accumulates.

On the surface, the assumption that the same orthographic representation is involved in lexical decision and the same–different task may seem at odds with the fact that the pattern of priming observed here in Experiment 1 and 2 were very different. Note however that our theory of masked priming based on the Bayesian Reader (e.g., Kinoshita & Norris, 2009; Norris & Kinoshita, 2008) makes it clear that priming effects are not task-invariant. According to the Bayesian Reader, perception involves Bayesian inference based on the accumulation of noisy evidence, with the hypothesis for which evidence is accumulated being determined by the goal of the task. In the context of masked priming a second critical assumption is that the prime and target are processed as a single perceptual object, and evidence from both the prime and target continuously updates the posterior probability of the hypothesis required to perform the task.

In lexical decision, the reader’s task is to decide whether the input matches any item in the lexicon (i.e., it is “a word”). More specifically, the task is to decide whether the input is more likely to be a word than a nonword. The reader is assumed to continuously sample from the input so as to home in on a sufficiently small region of lexical space to determine whether the input is more likely to be a word than a nonword. Accordingly, the evidence needed to make a lexical decision is expected to depend on the distributional characteristics of the lexical space. Specifically, in Arabic, the lexical space is structured around the tri-consonantal root system and the word pattern reflecting the standard Semitic morphology. As mentioned earlier, the transposition of any two letters of any Arabic root generates a hit (i.e., another existing root) more than 50% of the time, and most importantly, it preserves the typical Semitic morphological structure with a clearly identifiable three letter pseudoroot interleaved with an existing word pattern. This then is why, unlike English and other European languages written in the Roman alphabet, TL priming effects are absent in Arabic in lexical decision. The pervasive morphological structure of Arabic means that, any point in lexical space will be dense. Consequently, as with high-N words in English, there is little scope for observing masked priming effects by orthographic neighbors.

The situation with the same–different match task is different. The decision, in this case, does not need to factor in information about all the words in the lexicon, only about the referent. As a consequence, the lexical status (whether the referent/target is a word or a nonword) does not come into play, and as originally demonstrated by Kinoshita and Norris (2009) with nonwords written in the Roman alphabet, reliable identity and TL priming effects are observed, as was found with the Arabic nonwords in the present Experiment 3. Similarly, in contrast to lexical decision, the density of the lexical space does not come into play; all the reader needs to do is accumulate enough evidence to compare the likelihood that the target has the same form as the referent with the likelihood that it is different. This decision is based on the same orthographic representation consisting of letter identity and letter position information that serves as the input to lexical access, but the same–different decision does not concern the words in the lexicon, only whether it matches the referent string, in terms of the letter identities contained in the referent string and their order. Thus our results showing robust TL priming effects for Arabic words and nonwords in the same–different task, and contrary to the claim made by Perea et al. (2011), clearly point to the fact that the same–different match task taps the same—prelexical—level of representation in Arabic as it does in English or Hebrew, and that performance in this task is not modulated by morphological structure. Finally, at this prelexical level, the TL prime containing allographic variation (TL+Allog condition) provides greater certainty that the letter order is different from the referent/target string, resulting in reduced TL priming effects relative to the TL−Allog condition, that is, the modulatory effect of allographic variation in TL priming of Arabic words and nonwords.

We also note that the two aspects of the present study illustrate how cross-linguistic research contributes to the debate over a “universal model of reading,” spurred on by Frost (2012). Coltheart and Crain (2012) suggested that there is no property shared by all of the world’s writing systems. On the other hand, Kessler and Treiman (2015) pointed out that the fact that “writing is designed to be taken in by the eye leads to some characteristics that are essentially universal in modern writing” (p. 11). In this context, it may be noted that the majority of contemporary writing systems denote the order of letters in a linear spatial arrangement, regardless of whether it is written from left to right (as in English and other European languages), or right to left (as in Hebrew and Arabic) or vertically with logograms or logographs (as in Chinese or Japanese) the edge letter/logogram is the first letter/logogram, the adjacent letter is the second letter/logogram, and so on. Robust TL priming effects in the same–different task have now been reported for Arabic, as well as for many other languages including Hebrew, and European languages. This is consistent with our view (Norris & Kinoshita, 2012b) that TL priming effects originate in the uncertainty in the perception of letter order, due to the limits of precision in the perception of the spatial location of closely arranged objects. In contrast, the modulatory effect of allographic variation in TL priming observed in Experiments 2 and 3 sets Arabic apart from other languages. It is important to note that languages differ in many ways, and our results showed that it is not the Semitic morphology but the position-dependent allography in Arabic that is responsible for the modulation of TL priming effects in the same–different task. Taken together these two aspects of the results reinforce our view that in modeling orthographic processing, both language-universal (perceptual) processes and the exact nature of language-specific information used in the task need to be considered.

### Theoretical Implications

In this final section, we consider how current models of letter coding might accommodate the kind of allographic effects we have documented here. We first consider the spatial coding model SOLAR (Davis, 2010). On this view, the relative position of spatially distributed items is coded in terms of an activation gradient decreasing across letter position with the highest values being assigned to the initial letter of the string and the lowest to the last. In the Arabic word يسعدون *ysʕduun be happy*, the first letter يـ has the highest value, ـسـ the second value, ـعـ the third and so on until the final letter ن which will be assigned the lowest value. The model determines the degree of match between يسعدون and its TL−Allog prime يعسدون for instance by computing a Gaussian function that reflects letter position uncertainty. These Gaussian functions are then summed up to compute a superposition function whose amplitude will correspond to the match value. A large amplitude indicates a perfect match, whereas a low amplitude is a sign of a poor match value. One further characteristic of SOLAR is that the initial and final letters are explicitly tagged to capture the fact that they are more accurately coded than internal letters at least in Indo-European languages. In the context of Arabic, this initial-final letter tagging procedures will need to be expanded to tag all letter allographs as *initial*, *medial*, and *final* if the effects of allography in Arabic are to be captured. Given this modification, the spatial pattern to code the TL−Allog pair يعسدون-يسعدون will match perfectly because the transposed letters ـسـ and ـعـ will be tagged as medial allographs in both cases. The spatial pattern coding for the TL+Allog pair يسدعون-يسعدون, on the other hand, will have a lower match value because there is a mismatch between عـ initial in the prime and ـعـ medial in the target. A similar argument can apply to the substituted letter condition where a medial ـسـ is replaced by a medial ـشـ and a medial ـعـ by a medial ـغـ in the SL−Allog pair يشغدون-يسعدون thus preserving the overall shape of the word in the SL−Allog condition and leading to a higher match score than the SL+Allog pair يسزرون- يسعدون where the medial, but nonconnecting letter ـز and the final letter ر respectively, substitute for the medial letter ـعـ and the medial/final nonconnecting letter ـد. Thus tagging of letter allographs as initial, medial, final and isolated could in principle allow SOLAR to accommodate the presence of allographic effects in the same–different match task. What is less clear, however, is the ability of the model to capture the absence of TL-priming and allographic effects in the lexical-decision task. Future developments of the model will need to address this issue.

A second equally influential class of models is the open-bigram model (Grainger & van Heuven, 2003; Grainger & Whitney, 2004). In the version developed by Grainger and Van Heuven (2003), the model consists of three levels: An *alphabetic array*, which is a bank of detectors that simultaneously process all characters in the input and provides a retinotopic map of all characters in a given string. This information is then fed to the second level of the model, *the relative position map*, which consists of open bigrams that code the relative position of the letters in the string abstracting over surface form variations such as shape, size, and location of the stimulus. The final level of the model is the *orthographic word* where all words for which spelling is known are represented. On this account the TL−Allog prime يعسدون would have the open bigrams يعـ, يسـ, يد, و يـ, ن يـ, ـعسـ, ـعد, و ـعـ, ن ـعـ, ـسد, و ـسـ, ن ـسـ, دو, دن, نو and the TL+Allog prime يسدعون will have the open bigrams: يسـ, يد, عـ يـ, و يـ, ن يـ, ـسد, عـ ـسـ, و ـسـ, ن ـسـ, عـ ـد, ـدو, ـدن, عو, ن عـ, نو. Accordingly, each of them shares 14 out of its total 15 open bigrams with the target word ‘يسعدون’. Because the two types of primes show the same amount of open bigram overlap with the target, they should be equally effective on this account in facilitating the target word. This, as we saw above, is clearly not the case. The same holds true of the SL−Allog يشغدون and the SL+Allog يسزرون. With each of them sharing six of its 15 open bigrams with the target يسعدون, we are again led to expect comparable priming behaviors contrary to what we actually observe. In sum, although it is true that the model rightly predicts less priming in the substituted than the transposed letter conditions, it clearly falls short of predicting the modulatory effects of allography. One way around this would be to introduce an intermediate level of representation between the alphabetic array and the relative position map to capture allographic forms. On this scenario the TL−Allog prime يعسدون will share 14 open allographic bigrams with the target يسعدون, whereas the TL+Allog prime يسدعون will share only 9 such bigrams with the same target, rightly accommodating less facilitation in the latter condition. The same applies to the SL−Allog and the SL+Allog case where the introduction of an intermediate level of allographic representation will allow the model to capture the modulatory effect of surface from variation because the SL+Allog will share fewer of their allographic bigrams with the target than SL−Allog. In sum then, the introduction of an intermediate allographic level of representation allows the open bigram model to accommodate the present results. However, despite this modification, and just like SOLAR, the open bigram model remains unable to offer an account for why TL-priming effects can be made to come and go in Arabic depending on the task used.

The final family of models we consider is the class of models that assume noisy perception of letter order, including the overlap model (Gómez et al., 2008) and the noisy-slot Bayesian Reader (Norris, Kinoshita, & van Casteren, 2010) and the noisy channel (Norris & Kinoshita, 2012a) model. In itself, the idea that perception of order is noisy would be unable to explain the effect of allography. Allography should have no effect on the noise in the input. There must be some additional mechanism to take account of the constraint that allography places on letter order. In the noisy-channel Bayesian Reader, the model accumulates noisy samples from the input and uses them to construct a posterior distribution of item identity and order; which items are present, and in which order they appear in. An optimal Bayesian system should use all available perceptual information to determine letter identity and order. Allography provides a potentially important source of information to constrain the order in which letters are likely appear. That is, once allographic information becomes available, it should modify the prior expectation of the order of letters in the word. This conceptualization allows the Bayesian reader to naturally capture allographic effects in Arabic.

### Conclusions

The present paper provides evidence for the first time that TL-priming effects occur in Arabic and that they are modulated by allographic variation. The paper further demonstrates that these effects can be made to come and go depending on task demands. Collectively, these results pose a challenge to various letter-position coding models that explain TL priming effects in terms of specialized orthographic representations (e.g., SOLAR, open bigrams), but can be satisfactorily accommodated by models that assume the noisy perception of letter order (e.g., noisy slot Bayesian Reader). Future simulation work will need to be carried out to further refine our claims about orthographic coding schemes and their cross-linguistic implications.

## Figures and Tables

**Table 1 tbl1:** Arabic Script and Letter Groups

Groups	Example	Groups	Example
1	ب ت ث /b, t, θ/	5	س ش /s, ʃ/
2	ج ح خ /j, ℏ, x/	6	ص ض /s^ʕ^ d^ʕ^/
3	د ذ /d, ð/	7	ط ظ /t^ʕ^ ð^ʕ^/
4	ر ز /r, z/	8	ع غ /ʕ, γ/
9	ف ق ك ل م ن ه و ي أ /f, q, k, l, m, n, h, w, y, ʔ/		
*Note*. Stimuli are given in Arabic and in Buckwalter transliteration with the manipulated root letters in upper case.			

**Table 2 tbl2:** Fully and Partially Ligating Letters

Ligature	Initial	Medial	Final	Isolated	IPA
Full	غـ	ـغـ	ـغ/غ	غ	ɣ
Partial	د	ـد	ـد/د	د	d
*Note*. The Final letter column shows the allographs for the letter preceded by a nonligating and a ligating letter respectively.					

**Table 3 tbl3:** Sample Stimuli Used in Experiment 1

Stimuli	Word	Nonword
Prime		
1. Identity	يسعدون	انكهزت
	*ysʕduun*	*ʔ*nkhzt*
	‘be happy’	
2. TL−Allog	يعسدون	انهكزت
	*ysdʕuun	*ʔ*nhkzt*
3. TL+Allog	يسدعون	انكزهت
	*ysdʕuun	*ʔ*nkzht*
4. SL-Allog	يشغدون	انطعزت
	*yʃɣduun	*ʔ*nt*^ʕ^ʕ*zt*
5. SL+Allog	يسزرون	انكلطت
	*yszruun	*ʔ*nklt*^ʕ^*t*
6. Baseline	تتناثج	يتخاقم
	*ttnaaθj	*ytxaaqm
Target	يسعدون	انكهزت
	*ysʕduun*	*ʔ*nkhzt*
	‘be happy’	
*Note*. Examples are in Arabic script with a transliteration and an English gloss where appropriate (the asterisk indicates a nonword).		

**Table 4 tbl4:** Mean Lexical Decision Response Latencies (RT, in ms), Percent Error Rates (in Parentheses), and Magnitude of Facilitation (Priming in ms) in Experiment 1 (Lexical Decision Task)

Response and prime type	Word	Nonword
Identity	598 (.68)	737 (2.37)
TL−Allog	657 (.51)	763 (2.03)
SL−Allog	666 (1.53)	750 (3.39)
TL priming effect: SL−Allog minus TL−Allog	9	−13
TL+Allog	663 (1.01)	756 (2.88)
SL+Allog	678 (1.01)	750 (3.22)
Baseline	684 (1.19)	816 (2.03)
TL priming effect SL+Allog minus TL+Allog	15	−6

**Table 5 tbl5:** Sample Stimuli Used in Experiment 2

Stimuli	Same	Different
Reference	يسعدون	يخلدون
	*ysʕduun*	*yxlduun*
	‘be happy’	‘be eternal’
Prime		
1. Identity	يسعدون	يخلدون
	*ysʕduun*	*yxlduun*
	‘be happy’	‘be eternal’
2. TL−Allog	يعسدون	يلخدون
	*ysdʕuun	*ylxduun
3. TL+Allog	يسدعون	يخدلون
	*ysdʕuun	*yxdluun
4. SL−Allog	يشغدون	يظبدون
	*yʃɣduun	*yð^ʕ^*bduun*
5. SL+Allog	يسزرون	يخذكون
	*yszruun	*yxðkuun
6. Baseline	تتناثج	نلشدهم
	*ttnaaθj	*nlʃdhm
Target	يسعدون	ينتفعون
	*ysʕduun*	*yntfʕuun*
	‘be happy’	‘benefit’
*Note*. Examples are in Arabic script with a transliteration and an English gloss where appropriate (the asterisk indicates a nonword).		

**Table 6 tbl6:** Mean Response Latencies (RT, in ms), Percent Error Rates (in Parentheses), and Magnitude of Facilitation (Priming in ms) in Experiment 2 (Same–Different Task With Words)

Response and prime type	Same	Different
1. Identity	494 (5.07)	618 (5.67)
2. TL−Allog	486 (4.78)	613 (4.93)
3. SL−Allog	545 (4.63)	620 (4.48)
TL priming effect: SL−Allog minus TL−Allog	59	8
4. TL+Allog	517 (3.28)	614 (5.67)
5. SL+Allog	556 (3.43)	606 (4.33)
6. Baseline	570 (9.70)	627 (3.13)
TL priming effect SL+Allog minus TL+Allog	39	−8
*Note*. TL−Allog = Transposed Letter prime without allography; TL+Allog = Transposed Letter prime plus allography; SL−Allog = Substituted Letter prime without allography; SL+Allog = Substituted Letter prime plus allography.		

**Table 7 tbl7:** Experiment 3 Sample Nonword Stimuli in Arabic Script and Transliteration

Stimuli	Same	Different
Reference	انكهزت	تجغدون
	*ʔ*nkhzt*	*tjɣduun
Prime		
1. Identity	انكهزت	تجغدون
	*ʔ*nkhzt*	*tjɣduun
2. TL−Allog	انهكزت	تغجدون
	*ʔ*nhkzt*	*tɣjduun
3. TL+Allog	انكزهت	تجدغون
	*ʔ*nkzht*	*tjdɣuun
4. SL−Allog	انطعزت	تظفدون
	*ʔ*nt*^ʕ^*ʕzt*	*tð^ʕ^*fduun*
5. SL+Allog	انكلطت	تجنلون
	*ʔ*nklt*^ʕ^*t*	*tjnluun
6. Baseline	يتخاقم	ألعكها
	*ytxaaqm	*ʔ*lʕkhaa*
Target	انكهزت	انفشكن
	*ʔ*nkhzt*	*ʔ*nfʃkn*

**Table 8 tbl8:** Mean Response Latencies (RT, in ms), Percent Error Rates (in Parentheses), and Magnitude of Facilitation (Priming in ms) in Experiment 3 (Same–Different Task With Nonwords)

Response and prime type	Same	Different
1. Identity	539 (4.52)	620 (5.97)
2. TL−Allog	535 (5.00)	619 (7.58)
3. SL−Allog	582 (7.58)	626 (4.68)
TL priming effect: SL−Allog minus TL−Allog	47	8
4. TL+Allog	580 (6.45)	617 (6.45)
5. SL+Allog	*591 (6.45)*	608 (5.32)
6. Baseline	*596* (12.42)	620 (5.48)
TL priming effect SL+Allog minus TL+Allog	11	−9

**Table A1 tbl9:** Stimuli Used for the Word Response in Experiment 1 and the Same Response in Experiment 2

Target	TL−Allog	TL+Allog	SL−Allog	SL+Allog	Baseline
تنفذان tuNaF∼i*aani	تفنذان tuNaF∼i*aani	تنذفان tuNa*∼iFaani	تغبذان tuGaB∼i*aani	تندغان tuNaD∼iGaani	استقبش istaQBa$a
يشحذون ya$Ha*uuna	يحشذون ya$Ha*uuna	يشذحون ya$*aHuuna	يطخذون yaTXa*uuna	يشرلون ya$RaLuuna	تدزقين taDZuQiina
يجحدون yaJHaDuuna	يحجدون yaJHaDuuna	يجدحون yaJDaHuuna	يظلدون yaZLaDuuna	يجذقون yaJ*aQuuna	انصشفا inSaFa$aa
يستثمرون yastaVMiRuuna	يستمثرون yastaVMiRuuna	يستثرمون yastaVRiMuuna	يستخشرون yastaX$iRuuna	يستثحفون yastaVHiFuuna	انبدستما inBaDaStumaa
يتطلعان yataTaL∼aEaani	يتلطعان yataTaL∼aEaani	يتطعلان yataTaE∼Laani	يتغمعان yataGaM∼aEaani	يتطقزان yataTaQ∼aZaani	تثتئدون taVta > iDuuna
يحصدون yaHSuDuuna	يصحدون yaHSuDuuna	يحدصون yaHDuSuuna	يجقدون yaJQuDuuna	يحبزون yaHBuZuuna	أخلثها aXLaVuhaa
يتعهدون yataEaH∼aDuuna	يتهعدون yataHaE∼aDuuna	يتعدهون yataEaD∼aHuuna	يتغلدون yataGaL∼aDuuna	يتعثقون yataEaV∼aQuuna	تترنفين tatRaN∼aFiina
ينطلقان yanTaLiQaani	ينلطقان yanLaTiQaani	ينطقلان yanTaQiLaani	ينكفقان yanKaFiQaani	ينطخدان yanTaXiDaani	استكغمه istaKGaMahum
يسعدون yaSEaDuuna	يعسدون yaSEaDuuna	يسدعون yaSDaEuuna	يشغدون ya$GaDuuna	يسحجون yaSHaJuuna	تتناثج tataNaaVaJu
ينبذون yaNBu*uuna	يبنذون yaNBu*uuna	ينذبون yaN*uBuuna	يتفذون yaTFu*uuna	يندظون yaNDuZuuna	نهاشقك nuHaa$iQuka
يكنزون yaKNuZuuna	ينكزون yaKNuZuuna	يكزنون yaKZuNuuna	يجبزون yaJBuZuuna	يكشصون yaK$uSuuna	استجثر istaJVaRa
يقتصدون yaQtaSiDuuna	يصتقدون yaQtaSiDuuna	يقتدصون yaQtaDiSuuna	يختضدون yaXtaDiDuuna	يقتذشون yaQta*i$uuna	اشتكتطا i$taKataTaa
ينشدون yuN$iDuuna	يشندون yuN$iDuuna	يندشون yuNDi$uuna	يبسدون yuBSiDuuna	ينقجون yuNQiJuuna	تخفصان taXFiSaani
يتبخترون yataBaXTaRuuna	يتخبترون yataXaBTaRuuna	يتبرتخون yataBaRTaXuuna	يتقفترون yataQaFTaRuuna	يتبذتجون yataBa*TaJuuna	استكجطا istaKJaTaa
يحصلان yaHSuLaani	يصحلان yaHSuLaani	يحلصان yaHLuSaani	يجظلان yaJZuLaani	يحقظان yaHQuZaani	اجتزخت iJtaZaXat
يتفقدان yataFaQ∼aDaani	يتقفدان yataFaQ∼aDaani	يتفدقان yataFaD∼aQaani	يتعشدان yataEa$∼aDaani	يتفشعان yataFa$∼aEaani	تتفخجان tataFaX∼aJaani
ينظران yaNZuRaani	يظنران yaZaNuRaani	ينرظان yaNRuZaani	يجغران yaJGuRaani	ينلشان yaNLu$aani	نتطاكل nataTaaKaLu
يلحدون yuLHiDuuna	يحلدون yuLHiDuuna	يلدحون yuLDiHuuna	ينخدون yuNXiDuuna	يلرذون yuLRi*uuna	نتخاظش nataXaaZa$u
يحتجزان yaHtaJiZaani	يجتحزان yaHtaJiZaani	يحتزجان yaHtaZiJaani	يبتقزان yaBtaQiZaani	يحترطان yaHtaRiTaani	اشحظتني a$HaZtinii
يعطلان yuEaT∼iLaani	يطعلان yuTaE∼iLaani	يعلطان yuEaL∼iTaani	يثبلان yuVaB∼iLaani	يعرخان yuEaR∼iXaani	تفسشين tuFSi$iina
يفضلان yuFaDiLaani	يضفلان yuDaFiLaani	يفلضان yuFaLiDaani	يظبلان yuZaBiLaani	يفهجان yuFaHiJaani	انطذمت inTa*aMat
يسهرون yaSHaRuuna	يهسرون yaHSaRuuna	يسرهون yaSRaHuuna	يثكرون yaVKaRuuna	يسذلون yaS*aLuuna	انطفثت inTaFaVat
ينكرون yuNKiRuuna	يكنرون yuKNiRuuna	ينركون yuNRiKuuna	يشصرون yu$SiRuuna	ينصغون yuNSiGuuna	استبلظ istaBLaZa
يتطهرون yataTaH∼aRuuna	يتهطرون yataHaT∼aRuuna	يتطرهون yataTaR∼aHuuna	يتظخرون yataZaX∼aRuuna	يتطقبون yataTaQ∼aBuuna	انجحقتا inJaHaQataa
يشعوذان yu$aEWi*aani	يعشوذان yuEa$Wi*aani	يشوعذان yu$aWEi*aani	يسغوذان yuSaGWi*aani	يشبلذان yu$aBLi*aani	تجقحطون tuJaQHiTuuna
ينتقدون yaNtaQiDuuna	يقتندون yaNtaQiDuuna	ينتدقون yaNtaDiQuuna	يختثدون yaXtaViDuuna	ينترخون yaNtaRiXuuna	استلزئت istaLZa > at
يتعثرون yataEV∼aRuuna	يتثعرون yataEV∼aRuuna	يتعرثون yataER∼aVuuna	يتجطرون yataJT∼aRuuna	يتعزطون yataEZ∼aTuuna	انبخجتا inBXaJataa
يتحفزون yataHaF∼aZuuna	يتفحزون yataFaH∼aZuuna	يتحزفون yataHaZ∼aFuuna	يتلطزون yataLaT∼aZuuna	يتحدضون yataHaD∼aDuuna	اقتلرها aQtaLiRuhaa
يضطهدون yaDtaHiDuuna	يهطضدون yaHtaDiDuuna	يضطدهون yaDtaDiHuuna	يصطكدون yaStaKiDuuna	يضطقعون yaDtaQiEuuna	نستصمحه naStaSmiHuhu
ينقذون yuNQi*uuna	يقنذون yuQNi*uuna	ينذقون yuN*iQuuna	يحطذون yuHTi*uuna	ينكخون yuNKQiXuuna	تزثياه tuZViYaahu
ألهمنا aLHaManaa	ألمهنا aLMaHanaa	أهلمنا aHLaManaa	الضشنا aLaDa$naa	ادجمنا aDJaManaa	تندنقي tanDaNiQii
ألصقت aLSaQat	ألقصت aLQaSat	أصلقت aSLaQat	ألظغت aLDaGat	أرثقت aRVaQat	ينبتث yanBaTiVu
أفشلنا aF$aLnaa	أفلشنا aFLa$anaa	أشفلنا a$FaLanaa	أفسظنا aFSaZanaa	أرضلنا aRDaLanaa	يثجحون yaVHaJuuna
أشعلتها a$EaLathaa	أشلعتها a$LaEathaa	أعشلتها aE$aLathaa	أشقثتها a$QaVathaa	أفدلتها aFDaLathaa	يكتجحون yaKtaJiHuuna
أغضبني aGDaBanii	أغبضني aGBaDanii	أضغبني aDGaBanii	أغمشني aGMa$anii	أذلبني a*LaBanii	نتتابخ nataTaaBaXu
امتعضت iMtaEaDat	امتضعت iMtaDaEat	اعتمضت iEtaMaDat	امتشظت iMta$aZat	ابتدضت iBtaDaDat	يقفكون yaQFaKuuna
ألطمها aLTuMuhaa	ألمطها aLMuTuhaa	أطلمها aTLuMuhaa	ألحثها aLHuVuhaa	أدظمها aDZuMuhaa	يدغجهم yaDGaJuhum
ألفظها aLFuZuhaa	ألظفها aLZuFuhaa	أفلظها aFLuZuhaa	ألنضها aLNuDuhaa	أنزظها aNZuZuhaa	تجطهني taJTaHunii
أفعمته aFEaMtuhu	أفمعته aFMaEtuhu	أعفمته aEFaMtuhu	أفجظته aFJaZtuhu	أطزمته aTZaMtuhu	نحصمها naHSuMuhaa
أخفقوا aXFaQuu	أخقفوا aXQaFuu	أفخقوا aFXaQuu	أخمتوا aXMaTuu	أشذقوا a$*aQuu	تنثدين taNVuDiina
أظعنوا iZEaNuu	أظنعوا iZNaEuu	أعظنوا iEZaNuu	أظغجوا iZGaJuu	أزخنوا iZXaNuu	تنبثجا tanBaViJaa
أطعنه aTEaNuhu	أطعنه aTNaEuhu	أعطنه aETaNuhu	أطقله aTQaLuhu	أكذنه Ak*aNuhu	تظشكك taZ$uKuka
اعتصموا iEtaSaMuu	اعتمصوا iEtaMaSuu	اصتعموا iStaEaMuu	اعتشظوا iEta$aZuu	اشتدموا i$taDaMuu	نستطسبكي nastaTSiBukii
أنعشت aNEa$at	أنشعت aN$aEat	أعنشت aENa$at	أنغثت aNGaVat	أزكشت aZKa$at	تحيبا taHaY∼aBaa
ارتحلت iRtaHaLat	ارتلحت iRtaLaHat	احترلت iHtaRaLat	ارتثبت iRtaVaBat	ابتظلت iBtaZaLat	يجفضنا yaJFuDunaa
أعتقه aETaQahu	أعقته aEQaTahu	أتعقه aTEaQahu	أعنخه aENaXahu	أدغقه aDGaQahu	تحشخي taHa$∼aXii
أعشقها aE$aQuhaa	أعقشها aEQa$uhaa	أشعقها a$EaQuhaa	أعبخها aEBaXuhaa	أدثقها aDVaQuhaa	تلوهوا taLaW∼aHuu
اعتكفت iEtaKaFat	اعتفكت iEtaFaKat	اكتعفت iKtaEaFat	اعتجظت iEtaJaZat	أزتذفت iZta*aFat	نطيكها nuTaY∼iKuhaa
أضعفت aDEaFat	أضفعت aDFaEat	أعضفت aEDaFat	أضلبت aDLaBat	أبرفت aBRaFat	تظدست taZaD∼aSat
اغتبطت iGtaBaTat	اغتطبت iGtaTaBat	ابتغطت iBtaGaTat	اغتثنت iGtaVaNat	امتذطت iMta*aTat	نبزعها naBZuEuHaa
اغتصبوا iGtaSaBuu	اغتبصوا iGtaBaSuu	اصتغبوا iStaGaBuu	اغتطقوا iGtaTaQuu	اجتدبوا iJtaDaBuu	ينلشونه yaNLu$uunah
أغلقنا aGLaQnaa	أغقلنا aGQaLnaa	ألغقنا aLGaQnaa	أغشضنا aG$aSnaa	أزطقنا aZTaQnaa	نخدههم naXDaHuhum
أغمضت aGMaZat	أغضمت aGZaMat	أمغضت aMGaZat	أغلحت aGLaHat	أدزضت aDZaZat	نذشرك na*$uRuka
أبعثر uBaEViru	أبثعر uBaVEiru	أعبثر uEaBViru	أبقنر uBaQNiru	أبوغر uBaWGiru	ينزخه yaNZaXuhu
ارتجلت iRtaJaLat	ارتلجت iRtaJaLat	اجترلت iJtaRaLat	ارتنصت iRtaNaSat	اكتخلت IKtaJaLat	تتدائز tatDaa > aZu
ارتطمت iRtaTaMat	ارتمطت iRtaTaMat	اطترمت iTtaRaMat	ارتعكت iRtaEaKat	اهتصمت iHtaSaMat	نجاوكه nuJaaWiKuhu
ارتضينا iRtaDaYnaa	ارتيضنا iRtaYaDnaa	اضترينا iDtaRaYnaa	ارتخظنا iRtaXaTnaa	اختجينا iXtaJaYnaa	نجشتهم naJ$uTuhum
أغفيا aGFaYaa	أغيفا aGYaFaa	أفغيا aFGaYaa	أغمجا aGMaJaa	أركيا aRKaYaa	تجقره taJQuRuhu
اضغط وا iDGaTuu	اضطغوا iDTaGuu	اغضطو iGDaTuu	اضلقوا iDLaQuu	ادزطوا iDZaTuu	تنئزني taN>aZunii
أعسكر uEaSKiRu	أعكسر uEaKSiRu	أسعكر uSaEKiRu	أعلضر uEaLDiRu	أبزكر uBaZKiRu	نتثخد natVaX∼aDu
*Note.* Stimuli are given in Arabic and in modified Buckwalter transliteration with the manipulated root letters in upper case.					

**Table A2 tbl10:** Stimuli Used for the Nonword Response in Experiment 1

Target	TL−Allog	TL+Allog	SL−Allog	SL+Allog	Baseline
يعفذون yaEFuDuuna	يفعذون yaFEu*uuna	يعذفون yaE*uFuuna	يظبذون yaZBu*uuna	يعرزون yaERuZuuna	نلشدهم naL$aDuhum
يظهلان yuZaH∼iLAni	يهظلان yuHaZ∼iLAni	يظلهان yuZaL∼iHAni	يجضلان yuJaD∼iLaani	يظدزان yuZaD∼iZaani	احضكتي aHDaKaTii
يلهرون yuLHiRuuna	يهلرون yuHLiRuuna	يلرهون yuLRiHuuna	يصشرون yuS$iRuuna	يلجقون yuLJiQuuna	تستذنق tasta*NiQu
يتقثهرون yataQaHVaRuuna	يتقهثرون yataQaHVaRuuna	يتقثرهون yataQaVRaHuuna	يتجقبرون yataJaQBaRuuna	يتقثبحون yataQaVBaHuuna	تستأغفون tasta > GiFuuna
يسجعرون yuSaJEiRuuna	يسعجرون yuSaEJiRuuna	يسجرعون yuSaJRiEuuna	يسلثرون yuSaLViRuuna	يسجطكون yuSaJTiKuuna	تتقاجدن nataQaJDaNu
يغلرون yaGLaRuuna	يلغرون yaLGaRuuna	يغرلون yaGRaLuuna	يشظرون ya$ZaRuuna	يغبكون yaGBuKuuna	تطاعشنه iKEaa$un
يعطقرونه yuEaTQiRuunahu	يعقطرونه yuEaQTiRuunahu	يعطرقونه yuEaTRiQuunahu	يعكشرونه yuEaK$iRuunahu	يعطنظونه yuEaTNiZuunahu	تخلبزانه tuXaLBiZaanihi
يخكرها yaXKuRuhaa	يكخرها yaKXuRuhaa	يخركها yaXRuKuhaa	يضقرها yaDQuRuhaa	يخشتها yaX$uTuhaa	انحضخت inHaDaXat
تبقدجت taBaQDaJat	تقبدجت taQaBDaJat	تبدقجت taBaJaDat	تخفدجت taXaFDaJat	تبقحفت taBaQHaFat	يخلصفها yuXaLSiFuhaa
يتجكزون yuTaJKiZuuna	يتكجزون yutaJKiZuuna	يتجزكون yuTaJZiKuuna	يتبقزون yuTaBQiZuuna	يتجلظون yuTaJLiZuuna	أبردقهم uBaRDiQuhum
يتتبجذون yataTaBJa*uuna	يتتجبذون yataTaJBa*uuna	يتتبذجون yataTaB*aJuuna	يتتحقذون yataTaHQa*uuna	يتتبثكون yataTaBVaKuuna	أحفنطه uHaFNiJuhu
يجعسدون yuJaESiDuuna	يجسعدون yuJaSEiDuuna	يجعدسون yuJaEDiSuuna	يجطكدون yuJaTKiDuuna	يجعلبون yuJaELiBuuna	تبضدشان tuBADDi$aani
يتحنذج yataHaN*aJu	يتنحذج yataNaH*aJu	يتحذنج yataHa*NaJu	يتسبذج yataSaB*aJu	يتحنطج yataHaNTaJu	تنكلصان tuNaKLiSaani
يحقذلون yuHa*QiLuuna	يقحذلون yuQaH*iLuuna	يحذقلون yuHa*QiLuuna	يهصدلون yuHaSDiLuuna	يحقصطون yuHaQSiTuuna	أطفشبهم uTaF$iBuHum
تجتلظان taJtaLiZaani	تلتجظان taLtaJiZaani	تجتظلان taJtaZiLaani	تجتكفان taJtaKiFaani	تجتلران taJtaLiRaani	استقشغت istaQ$aGat
تغبزست taGaBZaSat	تبغزست taBaGZaSat	تغبسزت taGaBSaZat	تطهزست taTaHZaSat	تغفلست taGaFLaSat	يتكشطن yataKa$TaNu
يمتضلان yaMtaDiLaani	يضتملان yaMtaDiLaani	يمتلضان yaMtaLiDaani	يبتئلان yaBtaAiLaani	يمتشفان ya$taDiRaani	استظنجت istaZNaJat
يخلرون yaXLuRuuna	يلخرون yaLXuRuuna	يخرلون yaXRuLuuna	يهضرون yaHDuRuuna	يخضكون yaXDuKuuna	اشلهها a$LaHahaa
يضنجلان yaDaNJiLaani	ينضجلان yaNDaJiLaani	يضنلجان yaDaNLiJaani	يضقفلان yaDQaFiLaani	يضندذان yuDaNaDi*aani	تبسدغون tuBaSEiDuuna
يتعطزفون yataEaTZaFuuna	يتطعزفون yataTaEZaFuuna	يتعزطفون yataEaZTaFuuna	يتشحزفون yata$aHZaFuuna	يتعقلفون yataEaQLaFuuna	أكصنفها uKaSNiFuhaa
يتعكدتان yataEaKDataani	يتكعدتان yataKaEDataani	يتعدكتان yataEaDKataani	يتنضدتان yataNaDDataani	يتعلجتان yataEaLJataani	نقجلبهم nuQaJLiBuhum
تشلظان tu$LiZaani	تلشظان tu$LiZaani	تشظلان tu$ZiLaani	تجكحان tuJKiHaani	تشدزان tuSDiZaani	ارتخبي iRtaXibii
يفلدسون yuFaLDiSuuna	يلفدسون yuFaLDiSuuna	يفدلسون yuFaDLiSuuna	يقشدسون yuQa$DiSuuna	يفصطسون yuFaSTiSuuna	اجتدقتا iJtaDaQataa
يقلطرون yaQLaTiR∼uuna	يقطلرون yaQLaRiT∼uuna	يقلرطون yaQLaRiT∼uuna	يقصجرون yaQSaJiR∼uuna	يقلبضون yaQLaBiD∼uuna	ابتخطوا iBtaXaTuu
يقجطلان yuQaJTiLaani	يقطجلان yuQaTJiLaani	يقجلطان yuQaJLiTaani	يقعنلان yuQaENiLaani	يقجدنان yuQaJDiNaani	احزجدوا iHJaZiDuu
تقبظرون tuQaBZiRuuna	تقظبرون tuQaZBiRuuna	تقبرظون tuQaBZiRuuna	تقلجرون tuQaLJiRuuna	تقبهلون tuQaBHiLuuna	ابثطسو iBVaTiSuu
يتشنلغان yata$aNLaGaani	يتشلنغان yata$aLNaGaani	يتشنغلان yata$aNLaGaani	يتشحسغان yata$aHSaGaani	يتشندران yata$aNDaRaani	أكفرخهما uKaFXiRuhumaa
يكثحرون yaKVaHiR∼uuna	يثكحرون yaVKaHiR∼uuna	يكثرحون yaKVaHiR∼uuna	يكطجرون yaKTaJiR∼uuna	يكثفطون yaKVaFiT∼uuna	تتجلبدون tatJaLBaDuuna
يهفخلان yuHaFXiLaani	يهخفلان yuHaXFiLaani	يهفلخان yuHaFLiXaani	يهبشلان yuHaB$iLaani	يهفرزان yuHaFRiZaani	أشذمتاه a$*aMaTaahu
يبظزله yuBaZZiLuhu	يظبزله yuZaBZiLuhu	يبزظله yuBaZZiLuhu	يقثزله yuQaVZiLuhu	يبظقله yuBaZQiLuhu	أغسفقه uGaSFiQuhu
أعجقها uEaJ∼iQuhaa	أعقجها uEaQ∼iJuhaa	أجعقها uJaE∼iXuhaa	أعظصها uEaZ∼iSuhaa	أدزقها uDaZ∼iQuhaa	ينطكبه yanTaKiBuhu
أغلنها aGLuNuhaa	أغنلها aGNuLuhaa	ألغنها aLGuNuhaa	أعصثها aESuVuhaa	أرذنها aR*uNuhaa	تدظفان tuDaZ∼iFaani
أطلشت aTLa$at	أطشلت aT$aLat	ألطشت aLTa$at	أطظقت aTZaQat	أدذشت aD*a$at	تنقشس tanQa$iSu
أحلشها aHLu$uhaa	أحشلها aH$uLuhaa	ألحشها aLHu$uhaa	أحجطها aHJaTuhaa	أزقشها aZQu$uhaa	يحنصون yaHNiSuuna
ألطنها uLaT∼iNuha	ألنطها uLaN∼iTuha	أطلنها uTa∼LiNuha	ألظبها uLaZ∼iBuha	أحذنها uHa*∼iNuha	يأسجهم ya > SuJuhum
أعصظهم uEaS∼iZuhum	أعظصهم uEaZ∼iSuhum	أصعظهم uSaE∼iZuhum	أعكئهم uEak∼i > uhum	أزرظهم uZaR∼iZuhum	تيكلن tuYaKLinu
الضقوا iLDaQuu	القضوا iLDaQuu	اضلقوا iDLaQuu	الطغوا iLTaGuu	ادرقوا iDRaQuu	نجزظه naJZuZuhu
أعشجها aE$uJuhaa	أعجشها aEJu$uhaa	أشعغها a$EuGuhaa	أعظقها aEZuQuhaa	أذدغها a*DuGuhaa	يتلفطن yataLaF∼aTna
ألنثهم uLaNiVuhum	ألثنهم uLaViNuhum	أنلثهم uNaLiVuhum	ألظجهم uLaZiJuhum	أدعثهم aDEaVuhum	نجتغطه naJtaGiTuhu
أغنظها uGaN∼iZuhaa	أغظنها uGaZ∼iNuhaa	أنغظها uNaG∼iZuhaa	أغفعها uGaF∼iEuhaa	أردظها uRaD∼iZuhaa	تشأثون ta$>aVuuna
أغطنه uGaT∼iNuhu	أغنطه uGaN∼iTuhu	أطغنه uTaG∼iNuhu	أغجبه uGaJ∼iBuhu	أثدنه uVaD∼iNuhu	ندذره naD*uZuhu
أشلصتها a$LaStuhaa	أشصلتها a$SaLtuhaa	ألشصتها AL$aStuhaa	أشكعتها a$KaEtuhaa	أدقصتها aDQaStuhaa	نقفذهم naQFu*Uhum
أظلشها aZLu$uhaa	أظشلها aZ$uLuhaa	ألظشها aLZu$uhaa	أظبضها aZBuDuhaa	أدقشها aDQu$uhaa	تدذجون taD*uJuuna
أظغشت aZGa$at	أظشغت aZ$aGat	أغظشت aGZa$at	أظبفت aZBaFat	أحزشت aHZa$at	تهاذقت taHaa*Qat
أعطظت aETaZat	أعظطت aEZaTat	أطعظت aTEaZat	أعكنت aEKaNat	أزمظت aZMaZat	ينثخون yaNVuXuuna
أغطجوا aGTiJuu	أغجطوا aGJiTuu	أطغجوا aTGiJuu	أغلصوا aGLiSuu	أذغجوا a*GiJuu	تلاظصت taLaaZaSat
أغثلهم aGVuLuhum	أغلثهم aGLuVuhum	أثغلهم aVGuLuhum	أغشصهم aG$uSuhum	أزرلهم aZRuLuhum	يبحدون yuBHiDuuna
أعشضني aE$aDanii	أعضشني aEDa$anii	أشعضني a$EaDanii	أعصكني aESaKanii	أدرضني aDRaDanii	يكخطها yakXuTuhaa
أطغضت aTGaZat	أطضغت aTZaGat	أغطضت aGTaZat	اطثجت aTVaJat	اقزضت aQZaDat	نسئشه nuS > i$uhu
أكلطها aKLaTuhaa	أكطلها aKTaLuhaa	ألكطها aLKaTuhaa	أكعشها aKEa$uhaa	أبزطها aBZuTuhaa	يبشدون yaB$uDuuna
أشعضنا a$EaDna	أشضعنا a$DaEna	أعشضنا aE$aDna	اشخجنا a$XaJna	ادخضنا aDXaDna	تكجطين taKJuTiina
أهلظها aHLuDuhaa	أهظلها aHDuLuhaa	ألهظها aLHuDuhaa	أهجقها aHJuQuhaa	أزدظها aZDuZuhaa	يتجنوق yat∼aJiNuuQa
أعثبمها uEaVBiMuhaa	أعبثمها uEaBViMuhaa	أثعبمها uVaEBiMuhaa	أعجبمها uEaJBiMuhaa	أقذبمها uQa*BiMuhaa	أقذبمها yaSaBRiJaani
أعثمها aEVaMuhaa	أعمثها aEMaVuhaa	أثعمها aVEaMuhaa	اعطجها aETaJuhaa	اذرمها a*RaMahaa	نجافته nuJaaFiTuhu
أيلخوا aYLaXuu	أيخلوا aYXaLuu	أليخوا aLYaXuu	ايطكوا aYTaKuu	احذخوا AH*aXuu	تحدزين taHDuZiina
أعجصها aEJuSuhaa	أعصجها aESuJuhaa	أجعصها aJEuSuhaa	أعمغها aEMuGuhaa	أزرصها aZRuSuhaa	نقالثهم nuQaaLiVuhum
ارتضنت iRtaDaNat	ارتنضت iRtaNaDat	اضترنت iDtaRaNat	ارتقجت iRtaQaJat	اقتشنت iQta$aNat	يلادبه yaLaaDiBuhu
أضعقها aDEaQuhaa	أضقعها aDQaEuhaa	أعضقها aEDaQuhaa	اضكجها aDKaJuhaa	اطزقها aTZuQuhaa	نظاقبه nuZaaQiBuhu
أعلثهم uEaLiVuhum	أعثلهم uEaViLuhum	ألعثهم uLaEiVuhum	اعفضهم uEaFiDuhum	ازقثهم uZaQiVuhum	تحسثت taHaS∼aVat
أغنطت aGNaTat	أغطنت aGTaNat	أنغطت aNGaTat	أغسهت aGSaHat	أوزطت aWZaTat	تمظبوا tuMZiBuu
*Note.* In the appendices, the asterisk (*) represents the Arabic letter ذ and the tilde (∼) represents gemination.					

**Table B1 tbl11:** Stimuli Used for the Different Response in Experiment 2

Target	TL−Allog	TL+Allog	SL−Allog	SL+Allog	Baseline	Reference
يخلدون yaXLuDuuna	يلخدون yaLXuDuuna	يخدلون yaXDuLuuna	يظبدون yaZBuDuuna	يخذكون yaX*uKuuna	نلشدهم naLDa$uhum	ينتفعون yaNtaFiEuuna
يسهلان yaSaH∼iLAni	يهسلان yaHaS∼iLaani	يسلهان yuSaL∼iHaani	يجضلان yuJaD∼iLaani	يسدذان yuSaD∼i*aani	احضكتي aHDaKaTii	أغلبهم aGLuBuhum
يظهرون yuZHiRuuna	يهظرون yuZHiRuuna	يظرهون yuZRiHuuna	يصشرون yuS$iRuuna	يظزبون yuZZiBuuna	تستذنق tasta*NiQu	أهربوا uHRuBuu
يتجمهرون yataJaMhaRuuna	يتمجهرون yataMaJhaRuuna	يتجرهمون yataJaRhaMuuna	يتقبهرون yataQaBhaRuuna uuna	يتجصهكون yataJaShaKuuna	تستأغفون tasta<GiFuuna	تنبطحان tanBaTiHaani
يسيطرون yuSaYtiRuuna	ييسطرون yuYaStiRuuna	يسرطيون yuSaRtiYuunaa	يلثطرون yuLaVtiRuuna	يسكطهون yuSaKtiHuuna	تتقاجدن nataQaJDaNu	أزعم aZEumu
يصهرون yaSHaRuuna	يهصرون yaHSaRuuna	يصرهون yaSRaHuuna	يشظرون ya$ZaRuuna	يصبكون yaSBaKuuna	تطاعشنه iKEaa$un	أضحكني aDHaKanii
يعنصرونه yuEaNsiRuunahu	ينعصرونه yuNaEsiRuunahuahu	يعرصنونه yuEaRsiNuunahuah	يغطصرونه yuGaTsiRuunahuahu	يعجصظونه yuEaJsiZuunahuuunahu	تخلبزانه tuXaLBiZaanihi	تطفؤون tuTFi > uuna
ينثرها yaNVuRuhaa	يثنرها yaVNuRuhaa	ينرثها yaNRuVuhaa	يضقرها yaDQuRuhaa	ينشتها yaN$uTuhaa	انحضخت inHaDaXat	تتطفلان tataTaFaLaani
تبهرجت taBaHRaJat	تهبرجت taHaBRaJat	تبرهجت taBaRHaJat	تخفرجت taXaFRaJat	تبحفجت taBaHFaJat	يخلصفها yuXaLSiFuhaa	يؤمنونه yu<aM∼iNuunah
يتلفزون yuTaLfiZuuna	يلتفزونyuLaTfiZuunaa	يتزفلون yuTaZfiLuuna	يبقفزون yuBaQfiZuuna	يترفجون yuTaRfiJuuna	أبردقهم uBaRDiQuhum	تبينون tuBayYiNuuna
يتتلمذون yataTaLMa*uuna	يتلتمذون yataLaTMa* unana	يتتذملون yataTa*MaLuuna	يتبجمذون yataBaJMa*uuna	يتترمقون yataTaRmaQuuna	أحفنجه uHaFNiJuhu	أحركك uHaR∼iKuka
يجلمدون yuJaLmiDuuna	يلجمدون yuLaJmiDuuna	يجدملون yuJaDmiLuuna	يحقمدون yuHaQmiDuuna	يجرمقون yuJaRmiQuuna	تبضدشان tuBADDi$aani	يلبسها yaLBaSuha
يتحشرج yataHa$RaJu	يتشحرج yata$aHRaJu	يتحرشج yataHRa$aJu	يتسثرج yataSaVRaJu	يتحطلج yaTaHaTLaJu	تنكلصان tuNaKLiSaani	انقبضت inQaBaDat
يحمدلون yuHaMDiLuuna	يمحدلون yuMaHDiLuuna	يحدملون yuHaDMiLuuna	يهصدلون yuHaSDiLuuna	يحصطلون yuHaSTiLuuna	أطفشبهم uTaF$iBuHum	اصرخوا iSRaXuu
تختلفان taXtaLiFaani	تلتخفان taXtaLiFaani	تختفلان taXtaFiLaani	تجتكفان taJtaKiFaani	تختدقان taXtaDiQaani	استقشغت istaQ$aGat	يؤلفها yuaL∼iFuhaa
تغطرست taGaTRaSat	تطغرست taTaGRaSat	تغرطست taGaTRaSat	تطغرست taTaGRaSat	تغفلست taGaFLaSat	يتكشطن yataKa$TaNu	استأثرت istaVaRatu
يشتغلان ya$taGiLaani	يغتشلان yaGta$iLaani	يشتلغان ya$taLiGaani	يبتضلان yaBtaDiLaani	يشتدران ya$taDiRaani	استظنجت istaZNaJat	اقتتلوا iQtaTaLuu
يسترون yaSTuRuuna	يتسرون yaTSuRuuna	يسرتون yaSRuTuuna	يهضرون yaHDuRuuna	يسضكون yaSDuKuuna	اشلهها a$LaHahaa	يصدقه yuSaD∼iQuhu
يضمحلان yaDMahiLaani	يمضحلان yaMDahiLaani	يضلحمان yaDLahiMaani i	يصبحلان yaSBahiLaani	يضدحبان yaDDahiBaani	تبسدغون tuBaSEiDuuna	تتلهفان tataLaH∼aFaani
يتعجرفون yataEaJRaFuuna	يتجعرفون yataJaERaFuuna	يتعرجفون yataEaRJaFuuna	يتشحرفون yata$aHRaFuuna	يتعقلفون yataEaQLaFuuna	أكصنفها uKaSNiFuhaa	أثبتناها aVBaTnaahaa
يتعفرتان yataEaFRataani	يتفعرتان yataFaERataani	يتعرفتان yataEaRFataani	يتنضرتان yataNaDRataani	يتعلجتان yataEaLJataani	نقجلبهم nuQaJLiBuhum	تغتسلون taGtaSiLuuna
تصلحان tuSLiHaani	تلصحان tuSLiHaani	تصحلان tuSHiLaani	تجكحان tuJKiHaani	تصدزان tuSDiZaani	ارتخبي iRtaXibii	أعجزني aEJaZanii
يفهرسون yuFaHRiSuuna	يهفرسون yuHaFRiSuuna	يفرهسون yuFaRHiSuuna	يقشرسون yuQa$RiSuuna	يفصطسون yuFaSTiSuuna	اجتدقتا iJtaDaQataa	أسمعناه aSMaEnaahu
يقشعرون yaQ$aEiR∼uuna	يشقعرون ya$QaEiR∼uuna	يقرعشون yaQRaEi$∼uuna	يفسعرون yaFSaeiR∼uuna	يقدعطون yaQDaEiT∼uunana	ابتخطوا iBtaXaTuu	أنفخها aNFuXuhaa
يقنبلان yuQaNBiLaani	ينقبلان yuNaQBiLaani	يقلبنان yuQaLBiNaani	يفتبلان yuFaTbiLaani	يقدبذان yuQaDbi*aani	احزجدوا iHJaZiDuu	اختلسوا iXtaLaSuu
تقنطرون tuQaNTiRuuna	تنقطرون tuNaQTiRuuna	تقرطنون tuQarTiNuuna	تفبطرون uFaBtiRuuna	تقهطجون tuQaHtiJuuna	ابثطسوا iBVaTiSuu	أجشهت aJHa$at
يتشقلبان yata$aQLaBaani	يتشلقبان yata$aLQaBaani	يتشقبلان yata$aQBaLaani	يتشحسبان yata$aHSaBaani	يتشقدران yata$aQDaRaani	أكفرخهما uKaFXiRuhumaa	تزحلقت taZaHLaqat
يكفهرون yaKFaHiR∼uuna	يفكهرون yaFKaHiR∼uuna	يكرهفون yaKRahiF∼uuna	يطجهرون yaTJahiR∼uuna	يكدهمون yaKDahiM∼uuna	تتجلبدون tatJaLBaDuuna	انتصرن iNtaSaRna
يهيكلان yuHaYKiLaani	ييهكلان yuYaHkiLaani	يهلكيان yuHaLkiYaani	يمنكلان yuMaNkiLaani	يهركزان yuHaRkiZaani	أشذمتاه a$*aMaTaahu	يضطلعن yDtaLiEna
يبهدله yuBaHDiLuhu	يهبدله yuHaBDiLuhu	يبدهله yuBaDHiLuuna	يقثدله yuQaVDiLuhu	يبجقله yuBaJQiLuhu	أغسفقه uGaSFiQuhu	يتشاجرون yata$aaJaRna
أعبئها uEaB∼i<uhaa	أعئبها uEa<∼iBuhaa	أبعئها uBaE∼i<uhaa	أعظصها uEaZ∼iSuhaa	أدزئها uDaZ∼<ihaa > uhaa	ينطكبه yanTaKiBuhu	انفضوا iNFaDuu
أعجنها aEJuNuhaa	أعنجها aENuJuhaa	أجعنها aJEuNuhaa	أعصثها aESuVuhaa	أرذنها aR*uNuhaa	تدظفان tuDaZ∼iFaani	ارتبطت iRtaBaTat
أظلمت aZLaMat	أظملت aZMaLat	ألظمت aLZaMat	أظطقت aZTaQat	أدذمت aD*aMat	تنقشس tanQa$iSu	يتحيزون yataHaYyaZuuna
أسلكها aSLuKuhaa	أسكلها aSKuLuhaa	ألسكها aLSuKuhaa	أسجخها aSJuXuhaa	أزجكها aZJuKuhaa	يحنصون yaHNiSuuna	يتواعدون yataWaaEaDuuna
ألينها uLaY∼iNuha	ألنيها uYaL∼iNuha	أيلنها uYaL∼iNuha	ألظبها uLaZ∼iBuha	أطذنها uTa*∼iNuha	يأسجهم Ya<SuJuhum	اكتشفوا iKta$aFuu
أعطشهم uEaT∼i$uhum	أعشطهم uEaT∼i$uhum	أطعشهم uTaE∼i$uhum	أعكئهم uEaK∼i<uhumm	أزدشهم uZaD∼i$uhum	تيكلن tuYaKLinu	امتلكت iMtaLaKat
الهثوا iLHaVuu	الثهوا iLVaHuu	اهلثوا iHLaVuu	الطغوا iLTaGuu	ادرثوا iDRaVuu	نجزظه naJZuZuhu	تنجوان taNJuWaani
أعكسها aEKuSuhaa	أعسكها aESuKuhaa	أكعسها aKEuSuhaa	أعظقها aEZuQuhaa	أذدسها a*DuSuhaa	يتلفطن yataLaF∼aTna	يملئنه yaMLa<nahu
ألمعهم uLaMiEuhum	ألعمهم uLaEiMuhum	أملعهم uMaLiEuhum	ألظجهم uLaZiJuhum	أدصعهم uDaSiEuhum	نجتغطه naJtaGiTuhu	البثوا iLBaVuu
أغطسها uGaT∼iSuhaa	أغسطها uGaS∼iTuhaa	أطغسها uTaG∼iSuhaa	أغفعها uGaF∼iEuhaa	أدذسها uDa*∼iSuhaa	تشأثون ta$<aVuuna	أعدكم aEiDuKum
أغمسه uGaM∼iSuhu	أغسمه uGaS∼iMuhu	أمغسه uMaG∼iSuhu	أغجطه uGaJ∼iTuhu	أثدسه uVaD∼iSuhu	ندذره naD*uZuhu	يهبطون yaHbiTuuna
أجلستها aJLaStuhaa	أجسلتها aJSaLtuhaa	ألجستها aLJaStuhaa	أجكعتها aJKaEtuhaa	أدقستها aDQaStuhaa	نقفذهم naQFu*Uhum	تحسبون taHSiBuuna
أخلقها aXLuQuhaa	أخقلها aXQuLuhaa	ألخقها aLXuQuhaa	أخبضها aXBuDuhaa	أبدقها aBDuQuhaa	تدذجون taD*uJuuna	تستنبطون tastaNBiTuuna
أغطشت aGTa$at	أغشطت aG$aTat	أطغشت aTGa$at	أغثفت aGVaFat	أحزشت aHZa$at	تهاذقت taHaa*Qat	تخلصون tuXLiSuuna
أعتمت aETaMat	أعمتت aEMaTat	أتعمت aTEaMat	أعكنت aEKaNat	أزذمت aZ*aMat	ينثخون yaNVuXuuna	أرسمها arSuMuHaa
أعجموا uEJiMuu	أعمجوا uEMiJuu	أجعموا uJEiMuu	أعلظوا uELiZuu	أذغموا u*GiMuu	تلاظصت taLaaZaSat	أقدمهن uQaDdiMuhunna
أعضلهم aEDuLuhum	أعلضهم aELuDuhum	أضعلهم aDEuLuhum	أعشصهم aE$uSuhum	أزرلهم aZRuLuhum	يبحدون yuBHiDuuna	أصدروا aSDaRuu
أعطسني aETaSanii	أعسطني aESaTanii	أطعسني aTEaSanii	أعصكني aESaKanii	أذرسني aETaSanii	يكخطها yakXuTuhaa	أنميها uNaM∼iiha
أبغضت aBGaDat	ابضغت aBDaGat	اغبضت aGBaDat	ابثجت aBVaJat	اقزضت aQZaDat	نسئشه nuS<i$uhu	أوافقه uWaaFiQuhu
أسلخها aSLaXuhaa	أسخلها aSXaLuhaa	ألسخها aLSaXuhaa	أسبشها aSLaXuhaa	أبدخها aBDaXuhaa	تكجطين taKJuTiina	تلطخون tuLaT∼iXuuna
أنعمنا aNEaMna	أنمعنا aNMaEna	أعنمنا aENaMna	انخجنا aNXaJna	ادخمنا aDXaMna	يبشدون yaB$uDuuna	أبوا aBaWaa
أصلبها aSLuBuhaa	أصبلها aSBuLuhaa	ألصبها aLSuBuhaa	أصجقها aSJuQuhaa	أزدبها aSLuBuhaa	يتجنوق yataJaNWaQu	تنسكبان tanSaKiBaani
أعلقمها uEaLQiMuhaa	أعقلمها uEaQLiMuhaa	ألعقمها uLaEQiMuhaa	أعجبمها uEaJBiMuhaa	أبذقمها uBa*QiMuhaa	يصبرجان yaSaBRiJaani	أرعاها aREaahaa
أطبعها aTBaEuhaa	أطعبها aTEaBuhaa	ابطعها aBTaEuhaa	اطجمها aTJaMuhaa	اثرعها aVRaEuhaa	نجافته nuJaaFiTuhu	أحققها uHaQ∼iQuha
أثلجوا aVLaJuu	أثجلوا aVJaLuu	الثجوا aLVaJuu	اثطكوا aVTaKuu	احذجوا AH*aJuu	تحدزين taHDuZiina	ادنوا aDNuu
أعلفها aELuFuhaa	أعفلها aEFuLuhaa	ألعفها aLEuFuhaa	أعمغها aEMuGuhaa	أرزفها aRZuFuhaa	نقالثهم nuQaaLiVuhum	تكتئبان taKta<iBaani
ارتهنت iRtaHaNat	ارتنهت iRtaNaHat	اهترنت iHtaRaNat	ارتضشت iRtaDa$at	اقتشنت iQta$aNat	يلادبه yaLaaDiBuhu	أجهضت aJHaDat
أفعلها aFEaLuhaa	افلعها aFLaEuhaa	اعفلها aEFaLuhaa	افكجها aFKaJuhaa	ادزلها aDZaluhaa	نظاقبه nuZaaQiBuhu	أكلفهم uKaL∼iFuhum
أعظمهم uEaZiMuhum	اعمظهم uEaMiZuhum	اظعمهم uZaEiMuhum	اعفضهم uEaFiDuhum	ازقمهم aZQuMuhum	تمظبوا tuMZiBuu	أوجدوا aWJaDuu
أغلظت aGLaZat	أغظلت aGZaLat	ألغظت aLGaZat	أغسهت aGSaHat	أوبظت aWBaZat	تحسثت taHaS∼aVat	انهلوا iNHaLuu
*Note.* In the appendices, the asterisk (*) represents the Arabic letter ذ and the tilde (∼) represents gemination.						

**Table C1 tbl12:** Stimuli Used for the Same Response in Experiment 3

Target	TL−Allog	TL+Allog	SL−Allog	SL+Allog	Baseline
تنخذان tuNXi*aani	تخنذان tuXaNi*aani	تنذخان tuNa*∼iXaani	تجقذان tuJaQi*aani	تنظشان tuNaZi$aani	استنثل istaVNaLa
ينبحزون yanBaHiZuuna	ينحبزون yanHaBiZuuna	ينبزحون yanBaZiHuuna	ينتلزون yanTaLiZuuna	ينبكصون yanBaKiSuuna	تضاصموا taDaaSaMuu
يفشدان yuf$iDaani	يشفدان yu$FiDaani	يفدشان yufDi$aani	يقندان yuQNiDaani	يفئصان yuF<iSaani	انكرفتا inKaRaFataa
يبمزون yaBMuZuuna	يمبزون yaMBuZuuna	يبزمون yaBZuMuuna	يلخزون yaLXuZuuna	يبدجون yaBDuJuuna	تجامكن taJaaMaKna
تغلذان tuGLi*aani	تلغذان tuLaGi*aani	تغذلان tuG*iLaani	تبئذان tuB<i*aani	تغتحان tuGTiHaani	يكبهان yaKBaHaani
تنشكزن tan$aKiZna	تنكشزن tanKa$iZna	تنشزكن tan$aZiKna	تنئهزن tan<aHiZna	تنشظلن tan$aZiLna	تغاهمن taGaaMaHna
ينقبزان yanQaBiZaani	ينبقزان yanBaQiZaani	ينقزبان yanQaZiBaani	يجطزان yanJaTiZaani	ينقفمان yanQaFiMaani	تماجكوا taMaaJaKuu
يكبزون yaKBuZuuna	يبكزون yaBKuZuuna	يكزبون yaKZuBuuna	يطظزون yaTZuZuuna	يكنغون yaKNuGuuna	استمغق istaMGaQa
تلمران taLMuRaani	تملران taMLuRaani	تلرمان taLRuMaani	تهنران taHNuRaani	تلجفان taLJuFaani	تماسعن taMaaEaSna
يقظزون yaQZuZuuna	يظقزون yaZQuZuuna	يقزظون yaZZuQuuna	يفطزون yaFTuZuuna	يقبشون yaQBu$uuna	نستكلج nastaKLiJu
يصبذان yaSBu*aani	يبصذان yaBSu*aani	يصذبان yaS*uBaani	يئقذان ya<Qi*aani	يصطجان yaSTuJaani	تماعرا taMaaEaRaa
تشفدان ta$FuDaani	تفشدان taF$uDaani	تشدفان ta$DuFaani	تتثدان taTVuDaani	تشقمان ta$QuMaani	استنخش istaNXa$a
تطبذن taTBi*na	تبطذن taBTi*na	تطذبن taT*iBna	تجحذن tajHi*na	تطفخن taTFiXna	يكاعش yuKaaEi$u
يلظرون yaLZiRuuna	يظلرون yaZLiRuuna	يلرظون yaLRiZuuna	يسشرون yaS$iRuuna	يلقعون yaLQiEuuna	تنامكن TaNaaMaKna
يثعدان yaVEaDaani	يعثدان yaEVaDaani	يثدعان yaVDaEaani	يصضدان yaSDaDaani	يثجكان yaVJaKaani	انتغظا iNtaGaZaa
يقلزون yaQLIZuuna	يلقزون yaLQIZuuna	يقزلون yaQZILuuna	يعفزون yaEFIZuuna	يقتثون yaQTIVuuna	استظمش istaZMa$a
تظغوان taZGuWaani	تغظوان taGZuWaani	تغظوان taZWuGaani	تئثوان ta<VuWaani	تظكلان taZKuLaani	تكاخمن taKaaXaMna
يسلذون yaSLu*uuna	يلسذون yaLSu*uuna	يسذلون yaS*uLuuna	يهيذون yaHYu*uuna	يسبشون yaSBu$uuna	اظطمعا iZtaMaEa
تهكرون taHKiRuuna	تكهرون taKHiRuuna	تهركون taHRiKuuna	تئحرون ta<HiRuuna	تهبقون taHBiQuuna	تثاظمت taVaaZaMat
انكهزت inKaHaZat	انهكزت inHaKaZat	انكزهت inKaZaHat	انطعزت inTaEaZat	انكلضت inKaLaDat	يتخاقم yataXaaQaMu
انعمذا inEaMa*aa	انمعذا inMaEa*aa	انعذما inEa*aMaa	انغطذا inGaTa*aa	انعصشا inEaSa$aa	تواشلت taWaa$aLat
يلطران yaLTiRaani	يطلران yaTLiRaani	يلرطان yaLRiTaani	يصضران yaSDiRaani	يلظشان yaLZi$aani	استمجت iStaMaJat
انطلزا inTaLaZaa	انلطزا inLaTaZaa	انطزلا inTaZaLaa	انفغزا infaGaZaa	انطقفا inTaQaFaa	هاشعوا Haa$aEuu
انثقرن inVaQaRna	انقثرن inQaVaRna	انثرقن inVaRaQna	انثجرن inXaJaRna	انثنبن inVaBaVna	تصامفا taSaaFaMaa
يمتشدان yaMta$iDaani	يشتمدان ya$taMiDaani	يمتدشان yaMtaDi$aani	يصطندان yaStaNiDaani	يمتخجان yaMtaXiJaani	تقافسوا taQaaFaSuu
يتمزون yaTMuZuuna	يمتزون yaMTuZuuna	يتزمون yaTZuMuuna	يطخزون yaTXuZuuna	يتئشون ya<Ta$uuna	تقاطثن taQaaTaVna
استكطدت istaKTaDat	استطكدت istaTKaDat	استكدطت istaKDaTat	استبضدت istaBDaDat	استكنقت istaKNaQat	اعتقستا iEtaQaSataa
انشهزتا in$aHaZataa	انهشزتا inHa$aZataa	انشزهتا in$aZaHataa	انكلزتا inKaLaZataa	انشخفتا in$aXaFataa	تواغموا taWaaGaMuu
استخبدن istaXBaDna	استبخدن istaBXaDna	استخدبن istaXDaBna	استصبدن istaSQaDna	استخلكن istaXLaKna	انطدزوا inTaDaZuu
ينثجدان yanVaJiDaani	ينجثدان yanJaViDaani	ينثدجان yanVaDiJaani	ينظطدان yanZaTiDaani	ينثبنان yanVaBiNaani	انفقلتا inFaQaLataa
أعجقنا aEJaQnaa	أعقجنا aEQaJnaa	أجعقنا aJEaQnaa	أعبظنا aEBaZnaa	أدزقنا aDZaQnaa	يثطحون yaVaTaHuuna
أغلصوا aGLaSuu	أغصلوا aGSaLuu	ألغصوا aLGaSuu	أغئشوا aG<a$uu	أبذصوا aB*aSuu	يدخكان yaDXuKaani
أغمثتا aGMaVataa	أغثمتا aGVaMataa	أمغثتا aMGaVataa	أغكفتا aGFaKataa	أزرثتا aZRaVataa	يتهافط yatHaFaTu
أعبظن aEBaZna	أعظبن aEZaBna	أبعظن aBEaZna	أعنشن aENa$na	أزرظن aZRaZna	جاوخت JaaWaXat
أكغفت aKGaFat	أكفغت aKFaGat	أكغفت aGKaFat	أكصطت aKSaTat	أرطفت aRTaFat	تزاخق tuZaaXiQu
أعلظتا aELaZataa	أعظلتا aEZaLataa	ألعظتا aLEaZataa	أعنصتا aENaSataa	أدذظتا AD*aZataa	تكوجوا taKaWaJuu
أغصطوا aGSaTuu	أغطصوا aGTaSuu	أصغطوا aSGaTuu	أغشظوا aG$aZuu	أزدطوا aZDaTuu	استمخج istaMXaJa
أسعظتا aSEaZataa	أسظعتا aSZaEataa	أعسظتا aESaZataa	أسغطتا aSGaTataa	أدقظتا aDQaZataa	تجاغرت taJaaGaRat
أمغظوا aMGaZuu	أمظغوا aMZaGuu	أغمظوا aGMaZuu	أمجصوا aMJaSuu	أذزظوا a*ZaZuu	يخامهن yuXaaMiHna
أتغشا aTGa$aa	أتشغا aT$aGaa	أغتشا aGTa$aa	أتعثا aTEaVaa	أدزشا aDZa$aa	تراغث taRaaGaVat
أكعلوا aKEaLuu	أكلعوا aKLaEuu	أعكلوا aEKaLuu	أكغجوا aKGaJuu	أدذلوا AD*aLuu	استفقغ istaFQaGa
اعتقضوا iEtaQaDuu	اعتضقوا iEtaDaQuu	اقتعضوا iQtaEaDuu	اعتثموا iEtaVaMuu	ارتدضوا iRtaDaDuu	استغركت istaGRaKat
أعلثوا aELaVuu	أعثلوا aEVaLuu	ألعثوا aLEaVuu	أعبضوا aEBaDuu	أزدثوا aZDaVuu	يطامخن yaTaaMiXna
أصغفتا aSGaFataa	أصفغتا aSFaGataa	أغصفتا aGSaFataa	أصجطتا aSJaTataa	أرزفتا aRZaFataa	يتشاكخ yata$aaKaXu
اغتمكوا iGtaMaKuu	اغتكموا iGtaKaMuu	امتغكوا iMtaGaKuu	اغتنبوا iGtaNaBuu	اذتدكوا i*taDaKuu	استهفلت istaHLaFat
أشغستا a$GaSataa	أشسغتا a$SaGataa	أغشستا aG$aSataa	أشفكتا a$FaKataa	أذدستا a*DaSataa	تتعاظش tataEaZa$u
أعقثوا aEQaVuu	أعثقوا aEVaQuu	أقعثوا aQEaVuu	أعجخوا aEJaXuu	أرزثوا aRZaVuu	تكازرن taKaaZaRna
أنغفتا aNGaFataa	أنفغتا aNFaGataa	أغنفتا aGNaFataa	أنجصتا aNJaSataa	أدزفتا aDZaFataa	تماحجت taMaHaJat
أعفضوا aEFaDuu	أعضفوا aEDaFuu	أفعضوا aFEaDuu	أعقظوا aEQaZuu	أدزضوا aDZaDuu	استغثر istaGVaRa
أنغست aNGaSat	أنسغت aNSaGat	أغنست aGNaSat	أنثحت aNVaHat	أدذست AD*aSat	ضامجت DaaMaJat
أظعصتا aZEaSataa	أظصعتا aZSaEataa	أعظصتا aEZaSataa	أظطغتا aZTaGataa	أدزصتا aDZaSataa	استكفم IstaFKaMa
أبغموا aBGaMuu	أبمغوا aBMaGuu	أغبموا aGBaMuu	أبثجوا aBVaJuu	أدذموا AD*aMuu	يتنالس yataNaaLuSu
أشغنتا a$GaNataa	أشنغتا a$NaGataa	أغشنتا aG$aNataa	أشجفنتا a$JaFataa	أزذنتا AZ*aNataa	يتفاقت yataFaaQatu
اثتعسوا iVtaEaSuu	اثتسعوا iVtaSaEuu	اعتثسوا iEtavaSuu	اثتئنوا iVta<aNuu	ازدقسوا iZdaQaSuu	تنازدوا taNaaZaDuu
أمغشوا aMGa$uu	أمشغوا aM$aGuu	أغمشوا aGMa$uu	أمعتوا aMEaTuu	أرزشوا aRZa$uu	يخالزن yuXaaLiZna
أعكثنا aEKaVnaa	أعثكنا aEVaKnaa	أكعثنا aKEaVnaa	أعئضنا aE<aDnaa	أذدثنا a*DaVnaa	انهفقن iNHaFaQna
أعفتوا aEFaTuu	أعتفوا aETaFuu	أفعتوا aFEaTuu	أعصطوا aESaTuu	أردتوا aRDaTuu	تماكلت taMaaKaLat
أجغفن aJGaFnaa	أجفغن aJFaGnaa	أغجفن aGJaFnaa	أجعصن aJEaSnaa	أزدفن aZDaFnaa	يفاسع yuFaaSiEu
أطعقوا aTEaQuu	أطقعوا aTQaEuu	أعطقوا aETaQuu	أطغبوا aTGaBuu	أدذقوا AD*aQuu	استهشج istaH$aJa
أغتفتا aGTaFataa	أغفتتا aGFaTataa	أتغفتا aTGaFataa	أغصلتا aGSaLataa	أدقفتا aDQaFataa	تتهانط tataHaaNaTu
*Note.* In the appendices, the asterisk (*) represents the Arabic letter ذ and the tilde (∼) represents gemination.					

**Table C2 tbl13:** Stimuli Used for the Different Response in Experiment 3

Target	TL−Allog	TL+Allog	SL−Allog	SL+Allog	Baseline	reference
انصكدتا inSaKaDataa	انكصدتا inKaSaDataa	انصدكتا inSaDaKataa	انعشدتا inEa$aDataa	انصجفتا inSaJaFataa	يجابطون yaJaaBiTuuna	تتماجخ tataMaaJaXu
استعكوتا istaEKaWataa	استكعوتا istaKEaWataa	استعوكتا istaEWaKataa	استخثوتا istaXVaWataa	استعظجتا istaEZaJataa	يداغزان yuDaaGiZaani	تتجابض tataJaaBaDu
انفبزوا inFaBaZuu	انبفزوا inBaFaZuu	انفزبوا inFaZaBuu	انهقزوا inHaQaZuu	انفطكوا inFaTaKuu	يتغاسفن yataGaaSaFna	تطاقخن taTaaQaXna
ينقجران yanQaJiRaani	ينجقران yanJaQiRaani	ينقرجان yanQaRiJaani	ينخكران yanXaKiRaani	ينقفتان yanQaFiTaani	استمطغا istaMTaGaa	استفقش istaFQa$a
تسبدون tuSBiDuuna	تبسدون tuBSiDuuna	تسدبون tuSDiBuuna	تنئدون tuN<iDuuna	تسطفون tuSBTiFuuna	انشجقا in$aJaQaa	استفهز istaFHaZa
تكهوان taKHuWaani	تهكوان taHKuWaani	تكوهان taKWuHaani	تبخوان taBXuWaani	تكصمان taKSuMaani	عافلوا EaaFaLuu	تتفاسط tataFaaSaTu
انغفدوا inGaFaDuu	انفغدوا inFaGaDuu	انغدفوا inGaDaFuu	انصثدوا inSaVaDuu	انغطظوا inGaTaZuu	يتثابشن yataVaBa$na	تشاكثتا ta$aaKaVataa
استحكذت istaHKa*at	استكحذت istaKHa*at	استحذكت istaH*aKat	استنجذت istaNJa*at	استحصقت istaHSaQat	تغافطوا taGaaFaTuu	تجاقطوا taJaaQaTuu
ينطدان yaNTuDaani	يطندان yaTNuDaani	يندطان yaNDuTaani	يقضدان yaQDuDaani	ينظغان yaNZuGaani	انغوسا inGawaSaa	اجتظكت iJtaZaKat
انثقزوا inVaQaZuu	انقثزوا inQaVaZuu	انثزقوا inVaZaQuu	انضبزوا inDaBaZuu	انثشموا inVa$aMuu	يتواغرن yataWaGaRna	استفدخ istaFDaXa
انحشزا inHa$aZaa	انشحزا in$aHaZaa	انحزشا inHaZa$aa	انبلزا inBaLaZaa	انحقطا inHaQaTaa	مازغوا MaZaGuu	استخمف istaXMaFa
تنمزان taNMuZaani	تمنزان taMNuZaani	تنزمان taNZuMaani	تتظزان taTZuZaani	تنطخان taNTuXaani	تواجطن taWaaJaTna	تهافجتا taHaaFaJataa
تكطزان taKaTuZaani	تطكزان taTaKuZaani	تكزطان taKaZuTaani	تهظزان taHaZuZaani	تكجهان taKaJuHaani	يجطلون yaJTiLuuna	استوجض istaWJaSa
تغثدون taGViDuuna	تثغدون taVGiDuuna	تغدثون taGDiVuuna	تسخدون taSXiDuuna	تغمعون taGMiEuuna	أضجدني aDJaDanii	تفاثحا taFaaVaHaa
يهتتدون yaHtaTiDuuna	يتتهدون yaTataHiDuuna	يهتدتون yaHataDiTuuna	يحتبدون yaHtaBiDuuna	يهتفغون yaHtaFiGuuna	تلاكغان tuLaaKiGaani	استفشط istaF$aTa
نهبزها naHBuZuha	نبهزها naBHuZuha	نهزبها naHZuBuha	نتجزها naTJuZuha	نهئطها nuH<iTuhaa	يكعدون yaKEuDuuna	يسخمطن yasXaMiTna
تجغدون tuJGiDuuna	تغجدون tuGJiDuuna	تجدغون tuJDiGuuna	تظفدون tuZFiDuuna	تجنلون tuJNiLuuna	ألعكها aLEuKuhaa	يفاقجن yuFaaJiQna
يكثوون yaKVuWuuna	يثكوون yaVKuWuuna	يكوثون yaKWuVuuna	يخعوون yaXEuWuuna	يكفقون yaKFuQuuna	تغبذان taGBU*aani	يطشعون yaT$aEuuna
أستحلرهم astaHLiRuhum	أستلحرهم astaLHiRuhum	أستحرلهم astaHLiRuhum	أستشصرهم asta$SiRuhum	أستحبخهم astaHBiXuhum	يستنعظان yastaNEiZaani	تتثاحن tataVaHaNu
نمشزها naM$uZuha	نشمزها na$MuZuha	نمزشها naMZu$uha	نجحزها naJHuZuha	نمظطها naMZuTuha	أطعشنا aTEu$unaa	أثغشوا aVGaSuu
تثمزون tuVMiZuuna	تمثزون tuMViZuuna	تثزمون tuVZiMuuna	تظطزون tuZTiZuuna	تثقضون tuVQiDuuna	أغشعهم aG$aEuHum	انخبكت inXaBaKat
يعتذون yaETa*uuna	يتعذون yaTEa*uuna	يعذتون yaE*aTuuna	يقئذون yaQ<a*uuna	يعشصون yaE$aSuuna	نغئصها nuG<iSuhaa	تهالزن taHaaLaZna
أستسهذه astaSHi*uhu	أستهسذه astaHSi*uhu	أستسذهه astaS*iHuhu	أستطكذه astaTKi*uhu	أستسئكه astaS<iKuhu	يعتمصون yaEtaMiSuuna	اجتمقتا iJtaMaQataa
نجبزها nuJBiZuhaa	نبجزها nuBJiZuhaa	نجزبها nuJZiBuhaa	نغنزها nuGNiZuhaa	نجئثها nuJ<iVuhaa	أكظفها aKZuFuhaa	استهلج istaHLaJa
تصكرنا taSKuRunaa	تكصرنا taKSuRunaa	تصركنا taSRuKunaa	تهشرنا taH$uRunaa	تصبضنا taSBuDunaa	نجغظها naJGuZuha	أصحدتا aSHaDataa
يغصدون yaGSiDuuna	يصغدون yaSGiDuuna	يغدصون yaGDiSuuna	يثقدون yaVQiDuuna	يغئفون yaG<iFuuna	تصضنان taSDuNaani	هالدت HaaLaDat
نتكدهم naTKuDuhum	نكتدهم naKTuDuhum	نتدكهم naTDuKuhum	نلئدهم naL<aDuhum	نتبثهم naTBuVuhum	يغئضون yaG<iDuuna	يستخلم yastaXLiMu
تفلدون tuFLiDuuna	تلفدون tuLFiDuuna	تفدلون tuFDiLuuna	تخئدون tuX<iDuuna	تفنقون tuFNiQuuna	أهيطها uHaYiTuhaa	استطبك istaTBaKa
أستقجزهم astaQJiZuhum	أستجقزهم astaJQiZuhum	أستقزجهم astaQZiJuhum	أستمحزهم astaMHiZuhum	أستقئشهم astaQ<i$uhum	ييشونها yuYa$uNahaa	تماشخا taMaa$aXaa
يهكدان yuHKiDaaNi	يكهدان yuKHiDaaNi	يهدكان yuHDiKaaNi	يجشدان yuJ$iDaaNi	يهنخان yuHNiXaaNi	أخلشتها aXLa$tuhaa	يتضاخط yataZaaXaTu
أغظفوا aGZaFuu	أغفظوا aGFaZuu	أغظفوا aZGaFuu	أغثبوا aGBaVuu	أذلفوا a*LaFuu	تالجتا taaLaJaTaa	نتاخجهم nuTaaXiJuhum
اعتلصوا iEtaLaSuu	اعتصلوا iEtaSaLuu	التعصوا iLtaEaSuu	اعتبشوا iEtaBa$uu	ازدجصوا iZtaJaSuu	تنافجوا taNaaFaJuu	انفرتت inFaRaTat
أمعجتا aMEaJataa	أمجعتا aMJaEataa	أعمجتا aEMaJataa	أملظتا aMLaZataa	أرنجتا aRNaJataa	استمكح istaMKaHa	انفشكن inFaKa$na
أغفجوا aGFaJuu	أغجفوا aGJaFuu	أفغجوا aFGaJuu	أغئنوا aGAaNuu	أدظجوا aDZaJuu	تقامثت taQaaMaVat	استثكت istaVKaTa
أفغطوا aFGaTuu	أفطغوا aFTaGuu	أغفطوا aGFaTuu	أفجحوا aFJaHuu	أذمطوا a*MaTuu	يتخامث yataXaaMaVu	ماخسوا MaaXaSuu
اعستوا iESaTuu	اعتسوا iETaSauu	اسعتوا iSEaTuu	اعطضوا iETaDuu	ازحتوا iZHaTuu	يقافكن yuQaFiKna	ينثشون yaNVi$uuna
ألعضتا aLEaDataa	ألضعتا aLDaEataa	أعلضتا aELaDataa	ألغثتا aLGaVataa	أبرضتا aBRaDataa	انطمغا inTaMaGaa	تتخاكش tataXaaKa$u
أعثتوا aEVaTuu	أعتثوا aETaVuu	أثعتوا aVEaTuu	أعبخوا aEBaXuu	أشذتوا a$*aTuu	تجامقا taJaaMaQaa	يشاكطن yu$aaKiTna
أعتجتا aETaJataa	أعجتتا aEJaTataa	أتعجتا aTEaJataa	أعظغتا aEZaGataa	أدخجتا aDXaJataa	استثميا istaVMaYaa	تصابتن taSaaBaTna
أقعجوا aQEaJuu	أقجعوا aQJaEuu	أعقجوا aEQaJuu	أقبتوا aQEaJuu	أزرجوا aZRaJuu	تصامقن taSaaMaQna	تكامهن taKaaMaHna
أغفقت aGFaQat	أغقفت aGQaFat	أفغقت aFGaQat	أغصجت aGSaJat	أذزقت a*ZaQat	ضاكطن DaaKaTna	يستمشك yasTam$iKu
أغلشتا aGLa$ataa	أغشلتا aG$aLataa	ألغشتا aLGa$ataa	أغبفتا aGBaFataa	أبدشتا aBDa$ataa	تهافنا taHaaFaNaa	يخاوطن yuXaaWiTna
أغطثوا aGTaVuu	أغثطوا aGVaTuu	أطغثوا aTGaVuu	أغلحوا aGLaHuu	أردثوا aRDaVuu	انبلظا inBaLaZaa	اسطربوا isTaRaBuu
أعظقت aEZaQtu	أعقظت aEQaZtu	أظعقت aZEaQtu	أعبصت aEBaStu	أجزقت aJZaQtu	تشسفوا ta$aSa∼Fuu	تناسشت taNaaSa$at
أثغشوا aVGa$uu	أثشغوا aV$aGuu	أغثشوا aGVa$uu	أثبلوا aVBaLuu	أردشوا aRDa$uu	يتضاخط yatDaaXaTu	يمالشون yuMaaLi$uuna
أعقطنا aEQaTnaa	أعطقنا aETaQnaa	أقعطنا aQEaTnaa	أعكتنا aEKaTnaa	أزبطنا aZBaTnaa	يجابتن yuJaaBiTna	استظبكا istaZBaKa
أمغتوا aMGaTuu	أمتغوا aMTaGuu	أغمتوا aGMaTuu	أمئكوا aM<aKuu	أرزتوا aRZaTuu	يشكذون ya$Ku*uuna	استجمق istaJMaQa
أغهستا aGHaSataa	أغسهتا aGSaHataa	أهغستا aHGaSataa	أغجصتا aGJaSataa	أذئستا a*<aSataa	نشئزها nu$<iZuhaa	استثقه istaVaQaHu
اعتكنتا iEtaKaNataa	اعتنكتا iEtaNaKataa	اكتعنتا iKtaEaNataa	اعتلطتا iEtaLaTataa	ادتزنتا iDtaZaNataa	يطنذون yaTNu*uuna	استخمظن istaXMaZna
اسغكت aSaGaKat	اسكغت aSaKaGat	اغسكت aGaSaKat	اسجحت aSaJaHat	ازركت aZaRaKat	نلبخها naLBUXUhaa	استنتق istaNTaQa
التغجوا iLtaGaJuu	التجغوا iLtaJaGuu	اغتلجوا iGtaLaJuu	التصشوا iLtaSa$uu	اذتهجوا i*taHaJuu	ينتعفان yanTaEiFaani	يصاخم yuSaaXiMu
أعطثوا aETaVuu	أعثطوا aEVaTuu	أطعثوا aTEaVuu	أعبكوا aEBaKuu	أبدثوا aBDaVuu	يكفزون yaKFiZunna	استجخفنا istaJXaFnaa
أغصظتا aGSaZataa	أغظصتا aGZaSataa	أصغظتا aSGaZataa	أغسمتا aGSaMataa	أرزظتا aRZaZataa	تهبضان tuHBiDaani	يقتمجا yaQtaMiJaa
أعشتوا aE$aTuu	أعتشوا aETa$uu	أشعتوا a$EaTuu	أعنظو aENaZuu	أدذتوا AD*aTuu	يتنائزان yataNaa<aZaani	أبتهت aBTaHat
اكتعموا iKtaEaMuu	اكتمعوا iKtaMaEuu	اعتكموا iEtaKaMuu	اكتنصوا iKtaNaSuu	ادتزموا iDtaZaMuu	يحشنون yaH$uNuuna	يتساضكن yataSaaKaDna
أفغشنا aFGa$naa	أفشغنا aF$aGnaa	أغفشنا aGFa$naa	أفئطنا aF<aTnaa	أذزشنا a*Za$naa	تسقدان tuSQiDaani	يئتفدون yaAtaFiDuuna
أنعثها aNEuVuhaa	أنثعها aNVuEuhaa	أعنثها aENuVuhaa	أنبخها aNBuXuhaa	أدزثها aDZuVuhaa	يتفجدن yataFaJaDna	تضامشوا taDaaMa$uu
أغضلوا aGDaLuu	أغلضوا aGLaDuu	أضغلوا aDGaLuu	أغجقوا aGJaQuu	أدرلوا aDRaLuu	يثموون yaVMuWuuna	استقيفتا istaQyaFaTaa
اظتعست iZtaEaSat	اظتسعت iZtaSaEat	اعتظست iEtaZaSat	اظتبشت iZtaba$at	اذترست i*taRaSat	يبئصون yaB<iSuuna	تكامسوا taKaaMaSuu
أجغشتا aJGa$ataa	أجشغتا aJ$aGataa	أغجشتا aGJa$ataa	أجئضتا aJ<aDataa	أرذشتا AR*a$ataa	تعدظنا tuEDiZuna	ينكمخا yaNKaMiXaa
*Note.* In the appendices, the asterisk (*) represents the Arabic letter ذ and the tilde (∼) represents gemination.						

## Footnotes

1 In this task, masked priming effects are limited to the Same response and not observed with the Different response. Readers are referred to our previous works (e.g., Kinoshita & Norris, 2009, 2010; Norris & Kinoshita, 2008) for an explanation.

2 The asterisk is used to indicate a nonword.

3 In most languages the Roman upper-case letters can only appear as the first letter in a sentence or the first letter in a proper noun.

4 We compared this with the case in English by transposing the letters of 592 three-letter English words from the Celex database (Baayen, Piepenbrock, & Guilikers, 1995). Each base word gave rise to five possible permutations with a total of 2900 unique permutations (excluding repeats from items like *mum* and *pip*). Of these 2900 items, only 193 corresponded to existing words (e.g, *cat-act*; *apt-tap*). This means that letter transposition in English results in an existing word only 6.65% of the time, suggesting a lightly populated lexical space.

5 Because of a rank deficiency issue in the matrix of the fixed-effect model, the interaction term was not estimated.

## A

[Table-anchor tbl9][Table-anchor tbl10]

## B

[Table-anchor tbl11]

## C

[Table-anchor tbl12][Table-anchor tbl13]
